# HMGA1 promotes the progression of esophageal squamous cell carcinoma by elevating TKT-mediated upregulation of pentose phosphate pathway

**DOI:** 10.1038/s41419-024-06933-x

**Published:** 2024-07-30

**Authors:** Meng-Jie Liu, Yuan Zhao, Qiu-Tong Li, Xin-Yuan Lei, Kai-Yue He, Jin-Rong Guo, Jing-Yu Yang, Zhen-Hua Yan, Dan-Hui Wu, Lei Zhang, Yong-Ping Jian, Zhi-Xiang Xu

**Affiliations:** School of Life Sciences, Henan University, Kaifeng, Henan Province China

**Keywords:** Cancer metabolism, Cancer microenvironment, Cancer models, Oncogenes, Targeted therapies

## Abstract

Esophageal squamous cell carcinoma (ESCC) possesses a poor prognosis and treatment outcome. Dysregulated metabolism contributes to unrestricted growth of multiple cancers. However, abnormal metabolism, such as highly activated pentose phosphate pathway (PPP) in the progression of ESCC remains largely unknown. Herein, we report that high-mobility group AT-hook 1 (HMGA1), a structural transcriptional factor involved in chromatin remodeling, promoted the development of ESCC by upregulating the PPP. We found that HMGA1 was highly expressed in ESCC. Elevated HMGA1 promoted the malignant phenotype of ESCC cells. Conditional knockout of HMGA1 markedly reduced 4-nitroquinoline-1-oxide (4NQO)-induced esophageal tumorigenesis in mice. Through the metabolomic analysis and the validation assay, we found that HMGA1 upregulated the non-oxidative PPP. With the transcriptome sequencing, we identified that HMGA1 upregulated the expression of transketolase (TKT), which catalyzes the reversible reaction in non-oxidative PPP to exchange metabolites with glycolytic pathway. HMGA1 knockdown suppressed the PPP by downregulating TKT, resulting in the reduction of nucleotides in ESCC cells. Overexpression of HMGA1 upregulated PPP and promoted the survival of ESCC cells by activating TKT. We further characterized that HMGA1 promoted the transcription of TKT by interacting with and enhancing the binding of transcription factor SP1 to the promoter of TKT. Therapeutics targeting TKT with an inhibitor, oxythiamine, reduced HMGA1-induced ESCC cell proliferation and tumor growth. Together, in this study, we identified a new role of HMGA1 in ESCCs by upregulating TKT-mediated activation of PPP. Our results provided a new insight into the role of HMGA1/TKT/PPP in ESCC tumorigenesis and targeted therapy.

## Introduction

Esophageal squamous cell carcinoma (ESCC) and esophageal adenocarcinoma (EAC) are two major subtypes of esophageal cancer. The 5-year survival rate of esophageal cancer is less than 20%, which is mainly caused by the inability to diagnose at the early stage, high invasiveness of the tumor, and lacking of effective treatments [1–4]. There is an urgent need to find effective molecular targets for the treatment of esophageal cancer.

Metabolomics has become a new platform for biomarker discovery [5, 6]. Cell bioenergetics abnormality is a hallmark of cancer [7, 8]. To support tumor growth, cancer cells make a variety of metabolic adaptations. Metabolites not only act as substrates for the generation of bioenergetics, but also regulate biomass synthesis and gene expression of cancer cells [9]. For example, pentose phosphate pathway (PPP) generates pentose phosphate, such as ribose 5-phosphate (R5P), to provide substrates for nucleic acid synthesis and NADPH and GSH for the removal of reactive oxygen species (ROS) and generation of fatty acid within cells [10–13]. Thus, PPP plays a key role in meeting the needs of cancer cells for anabolism and resisting oxidative stress. Cancer cells directly or indirectly increase glucose flux to PPP [14]. Elevated PPP in cancer cells may thus distinguish them from normal cells. Targeting PPP could be a promising option for cancer treatment.

PPP is regulated by a variety of factors, such as tumor suppressors, oncoproteins, and intracellular metabolites [15]. Glucose-6-phosphate (G6P) dehydrogenase (G6PD) is a rate-limiting enzyme in oxidative PPP and determines the flux of G6P into the pathway [16, 17]. The transketolase (TKT) is a key enzyme in non-oxidative PPP [18, 19] and bidirectionally regulates carbon flux between non-oxidative PPP and glycolysis or gluconeogenesis. TKT has been shown to be upregulated in ovarian, breast, and prostate cancers [20–22]. However, how cancer cell metabolism is reprogrammed to increase the flux of glucose into PPP is largely unknown. In addition, the functional outcome of TKT upregulation in cancer remains to be elusive although TKT is reported to be highly expressed in esophageal cancer [23, 24].

High-mobility group AT-hook 1 (HMGA1) is a chromatin architectural transcriptional factor, which binds to the small groove of AT-rich DNA to change the chromatin structure and promote the assembly of transcriptional complexes, thus controlling the transcription of downstream effectors involved in basic cell processes, such as differentiation, transformation, and apoptosis. HMGA1 is highly expressed in multiple malignancies and correlates with poor prognosis of cancer patients [25–28]. However, the role of HMGA1 in ESCC remains unclear.

In the current study, we showed that HMGA1 expression was increased and positively correlated with disease progression in ESCC. Downregulation of HMGA1 suppressed the biological properties of ESCC cells. Overexpression of HMGA1 enhanced the binding of transcription factor specificity protein 1 (Sp1) to TKT promoter and promoted the expression of TKT, hence activating PPP to accelerate ESCC progression. HMGA1 knockdown suppressed PPP by downregulating TKT, resulting in a decrease in the synthesis of nucleotides, a reduction in DNA repair, and a persistence in DNA damage. Targeted inhibition of TKT with oxythiamine (OT) suppressed HMGA1-induced tumorigenesis of ESCCs. Our results provided a new therapeutic approach for the treatment of ESCC by inhibiting TKT-mediated PPP.

## Materials and methods

### Animal experiments

C57BL/6 male mice (6 weeks old) were purchased from the Animal Center of the National Science Council (Beijing, China). Mice were randomly assigned to subcutaneous injection with HMGA1 overexpression or control AKR cells (*n* = 6/each group). Single blind experiment was used for the animal observation. All animal procedures were approved by the institutional animal care and use committee (IACUC) at Henan University, China and complied with its guidelines. TKT inhibitor oxythiamine (OT) or PBS was applied into the mice by gavage every day and mice were euthanized two weeks later.

HMGA1^KI/KI^K14-cre^+^ conditional knock-in and HMGA1^flox/flox^K14 conditional knockout mice were originally ordered from Cyagen (Suzhou, China) and maintained in the laboratory for crossbreeding. 4-nitroquinoline-1-oxide (4NQO) dissolved in drinking water (80 mg/L) was used for the induction of esophageal cancer in mice. Continuous induction was performed for 5 months. The mice were weekly observed and murine status and body weight were recorded.

### Human tissue samples

ESCC tissue specimens from tumors and adjacent normal tissues were collected in Beiguan District, Anyang City, Henan Province. All patients were diagnosed with ESCC. Usage of the tissue was approved by Ethics Committee of Henan University.

### Cell culture and transfection

Human ESCC cell lines KYSE30 and TE13 were maintained in the lab. Murine ESCC cell line AKR was purchased from Otwo Biotech (Cat#: HTX2545; Shenzhen, China). All cell lines used for the experiments were authenticated and mycoplasma negative. Human ESCC cell lines, KYSE30, TE13 and murine ESCC cell line AKR were routinely cultured in RPMI-1640 medium (10-040-CV, Corning) and HEK 293T cells were cultured in DMEM medium (10-013-CV, Corning) supplemented with 10% fetal bovine serum (FBS, S711-011S, Lonsera) in 5% CO_2_ at 37 °C. Plasmids and small interfering RNAs (siRNAs) were transfected using Lipofectamine™ 2000 (Cat# 11668-019, Invitrogen) according to the manufacturer’s instructions.

### Antibodies and chemicals

Antibodies against HMGA1 (ab129153), Ki67 (ab16667), and TKT (ab112997) were purchased from Abcam (Cambridge, MA). Antibodies against γ-H2AX (sc-517348), HMGA1 (sc-393213), and TKT (sc-390179) and mouse IgG (sc-2025) were purchased from Santa Cruz Biotechnology (Santa Cruz, CA, USA). Antibodies against cleaved caspase 3 (9661S) and SP1 (9389S) and rabbit IgG (#2729) were purchased from Cell Signaling Technology (Danvers, MA). Antibody against γ-H2AX (AB3322) was purchased from Abways (Shanghai, China). Antibody against Bcl2 (12789-1-AP) was purchased from Proteintech (Wuhan, China). Anti-CDK4 (R23888) and anti-cyclin D1 (380999) antibodies were purchased from Zen-Bioscience Co., Ltd. (Chengdu, China). Oxythiamine (OT) was obtained from Sigma (Shanghai, China).

### Plasmid construction

HMGA1, SP1, and TKT overexpression plasmids were constructed based on V3-HA-Flag. Human HMGA1 and TKT cDNAs were amplified with PCR and then cloned into the lentivirus vector lentiCRISPR V3-Puro with an N-terminal Flag tag with restriction enzymes *Xho*I and *EcoR*I. TKT was also cloned into pcDNA3.1 to construct an overexpression plasmid. ShRNAs targeting HMGA1 and siRNAs targeting TKT were synthesized by GenePharma (Shanghai, China). The shRNA (siRNA) sequences of HMGA1 and TKT are provided in Table S1. To generate TKT promoter luciferase reporter plasmid, DNA fragment of human TKT gene promoter (−1903/+597) was amplified with PCR and sub-cloned into the *Mlu*I-*Xho*I sites of the pGL3-basic vector.

### Co-immunoprecipitation (co-IP) and western blot

Proteins were extracted from human ESCC cells using radio-immunoprecipiation assay (RIPA) lysis buffer in the presence of a PhosSTOP phosphatase inhibitor mixture (Roche) and a protease inhibitor mixture (Roche). Extracted proteins were quantitated using the BCA protein assay kit (Solarbio, China). For co-IP, 800 μg lysates were incubated with 5 μg antibody or IgG for overnight at 4 °C. The next day, 30 μl of Protein-A/G beads (sc-2003, Santa Cruz) were added and kept on a rotator for 2 h at 4 °C. The beads were washed with immunoprecipitation buffer 5 times at 4 °C and then mixed with 15 μl of 1× loading buffer for western blot analysis. Proteins were separated using 10–12% SDS-PAGE and transferred to PVDF membrane (Millipore, USA). The membranes were blocked with 5% bovine serum albumin (Solarbio, China) and probed with primary and second antibody.

### Quantitative real-time PCR

For quantitative real-time PCR (qRT-PCR) analysis, cDNA was synthesized with a HiScripIII RT SuperMix Kit for QPCR (R323-01, Vazyme, Nanjing, China). qRT-PCR was performed using ChamQ Universal SYBR qPCR Master Mix (Q711-02, Vazyme, Nanjing, China) [29]. Primer sequences of qRT-PCR were provided in Table S1.

### Chromatin immunoprecipitation (ChIP)

KYSE30/shNC and KYSE30/shHMGA1 cells were cross-linked using 1% formaldehyde and sonicated to obtain DNA fragments of ~700 base pairs (bp) [30]. After centrifugation, the supernatants were subjected to immunoprecipitation overnight with antibody against SP1 and then pulled down with Protein A/G for isolating the chromatin-antibody complexes [31]. The cross-linking was then reversed, and the precipitated DNA fragments were purified and analyzed by qPCR with the primers (TKT promoter) listed in Table S1.

### Cell cycle analysis

Cell cycle distribution was analyzed by PI (Calbiochem) staining. TE13 cells were treated with OT. Cells were fixed with 70% (vol/vol) ethanol for at least 1 h and incubated with 50 μg/mL PI for 30 min. The PI signal was measured by flow cytometry.

### Apoptosis analysis

TE13 cells were treated with OT for 24 h. Cells were then trypsinized and suspended in Annexin V binding buffer (BD Biosciences) containing Annexin V-FITC (MBL International) and PI (BD Biosciences) for 15 min at room temperature based on the manufacturers’ instructions. Samples were immediately analyzed with the flow cytometry.

### ROS analysis

To determine the level of ROS, an ROS Assay Kit (S0033S, Beyotime Biotechnology, China) was used to measure the content of ROS according to the manufacturer’s instructions and ROS production was measured by flow cytometry.

### NADPH, GSH, and TKT enzymatic assays

NADPH/NADP^+^ ratios were measured by a human NADPH ELISA Kit (YJ703492, Enzyme-linked Biotech) according to the manufacturer’s instructions. To analyze the GSH level, ESCC cells were seeded in 6-well plates and cultured for overnight. The cells were then exposed to TKT inhibitor, OT, for 8 h. The content of GSH in cells was determined using a human glutathione (GSH) ELISA Kit (BC1175, Solarbio, China) according to the manufacturer’s instructions. Following the transfection and treatment, TKT activity was tested using a TKT ELISA Kit (YJ711280) according to the manufacturer’s instructions.

### Luciferase assay

The promoter region of human TKT was amplified by PCR and inserted into the pGL3 vector. The reporter constructs containing various lengths of TKT promoter and their mutants were generated by subsequent PCR-based cloning. KYSE30 and 293T cells were plated in 96-well plates and then co-transfected with pGL3 constructs and Renilla A plasmid. After 48 h, the luciferase activity of the luciferin substrate was measured using the Duo-Lite Luciferase Assay System Kit (DD1205-01, Vazyme) [32].

### Immunohistochemistry

ESCC tissue specimens from both tumors and adjacent normal tissues were embedded in paraffin and sectioned at a thickness of 5 μm. Immunohistochemistry (IHC) was performed and analyzed as previously described [33].

### Proliferation assays

A density of 1 × 10^3^ cells per well were inoculated in 6-well plates and cultured at 37 °C in an incubator containing 5% carbon dioxide for 2 weeks. The cells were fixed with 4% paraformaldehyde for 30 min, washed with PBS for 3 times, and incubated with 0.5% crystal violet solution for another 30 min. Cells were then cleaned with purified water and colony formation was determined under a microscope. EdU incorporation assay was used to evaluate cell proliferation with a kit from Ribbo Biotech (Cat #: C10310-1). Then results were analyzed with a inverted fluorescence microscope (ZEISS Axio Observer7, Germany).

### Targeted metabolomic assay

KYSE30/shNC and KYSE30/shHMGA1 cells were extracted for metabolite assay. The extracts were analyzed using Shimadzu LC Nexera X2 UHPLC coupled with a QTRAP 5500 LC MS/MS (AB Sciex). Chromatographic separation was performed with ACQUITY UPLC UPLC BEH Amide analytic column (Metware, Wuhan, China) [34].

### Immunofluorescence assay

KYSE30 cells and TE13 cells were fixed in 4% paraformaldehyde for 20 min, permeabilized with 0.05% Triton X-100 in TBS for 30 min, and then blocked with 3% BSA for 1 h. Cells were then incubated with anti-γ-H2AX antibody (1:500) for overnight at 4 °C. The following day, cells were washed with washing buffer and incubated with the secondary antibody Alexa-488 (1:1000) (Abcam, ab150077, UK) for 1 h at room temperature. Nuclei were stained with DAPI (UE, Suzhou) for 10 min at room temperature. γ-H2AX fluorescent images were acquired with a laser scanning confocal microscope (ZEISS, LSM980, Germany) and analyzed with ZEN ImageJ software.

### Single-cell RNA-seq data analysis

ESCC single-cell RNA sequencing data (GSE188900) were downloaded from GEO database and re-analyzed using the Seurat software package to compare HMGA1 expression levels in different cell types in ESCC. The method for the analysis followed the protcol previously reported [35, 36].

### Statistics

Statistical analyses were performed using GraphPad Prism software (version 8). Statistical significance was defined as *p* < 0.05. All data were obtained from 3 independent experiments and are presented as the mean ± standard deviation (SD).

## Results

### HMGA1 is highly expressed in esophageal cancer

**Fig. 1:**
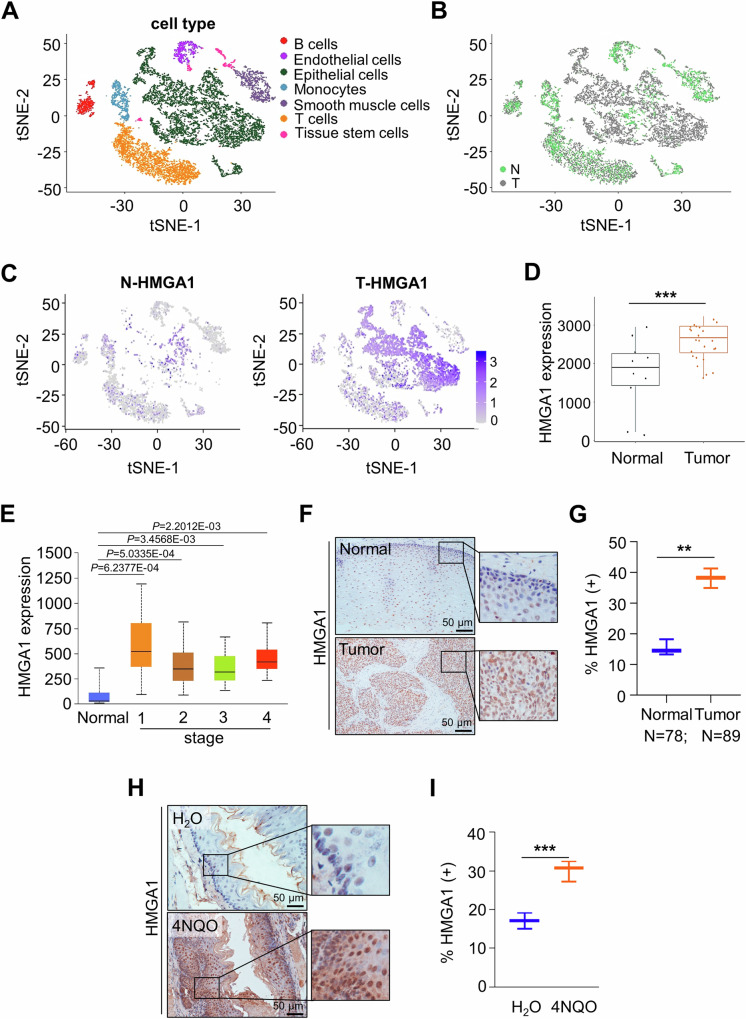
HMGA1 is highly expressed in esophageal cancer. **A**–**C** The tSNE map showed the distribution of epithelial cells from normal (N) and tumor (T) in esophageal tissue in ESCC patients from scRNA-seq data in the GEO database (GSE188900 dataset). HMGA1 was mainly expressed in epithelial cells from tumor (**C**). **D** HMGA1 expression in ESCC (*n* = 28) and paired paracancerous (*n* = 10) samples in the GEO database (GSE45670 dataset). **E** The TCGA database analyzes the expression of HMGA1 at normal and tumor tissues of different stages of ESCCs. **F**, **G** Representative IHC staining of HMGA1 in paired ESCC tissues. Scale bars: 50 μm. **H**, **I** Representative IHC staining of HMGA1 in 4NQO-induced esophageal tumor tissue and H_2_O-treated normal esophageal tissue. Scale bars: 50 μm.

To determine the involvement of HMGA1 in ESCC, we firstly analyzed single-cell RNA sequencing (scRNA-seq) data of ESCC from the GSE188900 dataset in the GEO database. Epithelial cells, immune cells, endothelial cells, and other tumor microenvironmental cells were identified and annotated with classical markers (Figs. 1A and S1). We screened data for highly heterogeneous genes in different cell subtypes and performed a t-distributed stochastic neighbor embedding (t-SNE) visualization to label cell clusters as normal and tumor cells (Fig. 1B, C). It showed that HMGA1 was principally expressed in malignant epithelial cells of the esophageal carcinoma tissues (Fig. 1C). To validate the finding that HMGA1 was highly expressed in ESCC in the scRNA-seq, we analyzed the ESCC data in the GSE45670 dataset in the GEO database, which includes 28 cases of ESCC and 10 cases of normal esophagus tissues. Levels of HMGA1 in ESCC tumors were markedly higher than those in normal esophageal tissues (Fig. 1D). To characterize the expression of HMGA1 in different stages of ESCCs, we analyzed the level of HMGA1 in primary tumors at various stages of ESCCs, and found that expression of HMGA1 in each stage of ESCC tumors was significantly higher than that in para cancerous tissues (Fig. 1E).

To determine the expression of HMGA1 in ESCC, we used IHC to stain HMGA1 in ESCC tumors and corresponding normal tissues. HMGA1 was only weakly expressed in the nucleus and in the cytoplasm in normal esophagus tissues (Fig. 1F, G). In contrast, HMGA1 was strongly expressed in the nucleus and weakly stained in the cytoplasm of esophageal cancer cells (Fig. 1F, G). Similar findings were obtained in esophageal tissues of mice treated with 4NQO for the induction of esophageal cancers demonstrating that HMGA1 was highly expressed in esophageal malignancies and rarely detected in adjacent normal esophageal tissues (Fig. 1H, I).

### Elevated HMGA1 promotes malignant phenotype of ESCC cells

**Fig. 2:**
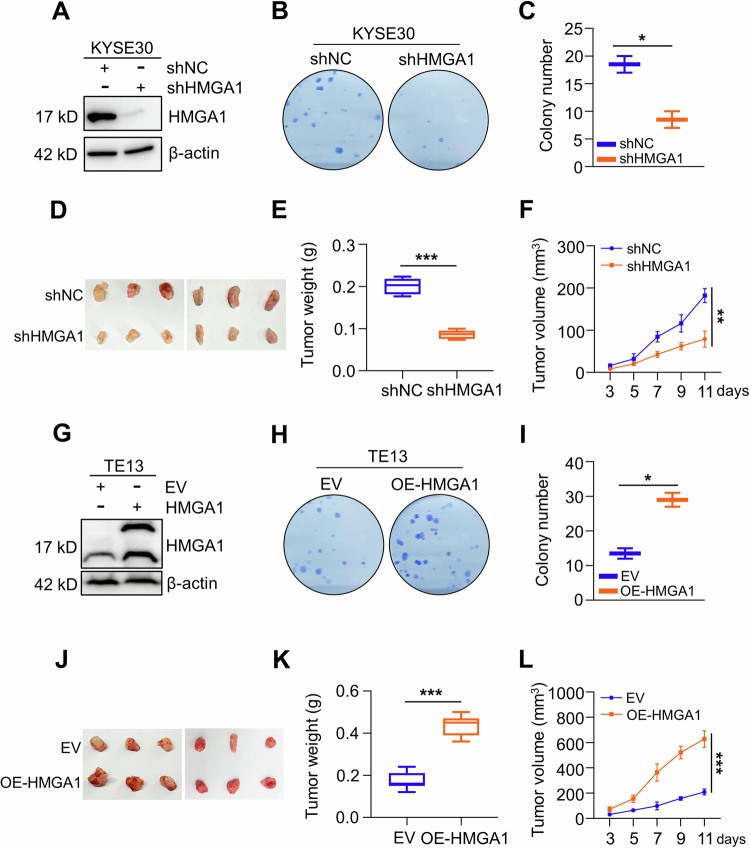
Elevated HMGA1 promotes malignant phenotype of ESCC cells. **A**–**C** HMGA1 knockdown inhibits the proliferation of ESCC cells. HMGA1 was knocked down in KYSE30 cells. Western blot was performed to detect the efficiency of the knockdown (**A**). Cell proliferation of the control and shHMGA1 cells was detected by colony formation assay (**B**). Colonies in (**B**) were calculated and shown in (**C**), *n* = 3. **D**–**F** Subcutaneous syngeneic tumors generated from HMGA1-knocked down mouse esophageal cancer AKR cells, *n* = 6. **D** Tumors, **E** Tumor weight, **F** Tumor volume. Tumor length (L) and width (W) were measured using vernier calipers every other day from day 3 after transplantation. The tumor volume was calculated using the formula (L × W^2^)/2 and presented as mean ± SEM. **G**–**I** HMGA1 overexpression promotes the proliferation of ESCC cells. A stabe cell line with HMGA1 overexpression was established in TE13 cells. Western blot was performed to detect the efficiency of the enforced expression (**G**). Cell proliferation of the control and HMGA1 overexpression cells was detected by colony formation assay (**H**). Colonies in (**H**) were calculated and shown in (**I**), *n* = 3. **J**–**L** Tumors and their weight and volume of subcutaneous syngeneic tumors generated from HMGA1-overexpressed mouse esophageal cancer AKR cells. Tumor establishment and measurement were performed as described in (**D**–**F**), *n* = 6. *P < 0.05, **P < 0.01, and ***P < 0.001.

To determine the role of HMGA1 in the maintenance of malignant phenotype of ESCC, we investigated the impact of HMGA1 manipulations on ESCC cell proliferation and tumor growth. Cell colony formation assay showed that knockdown of HMGA1 inhibited the proliferation of KYSE30 cells, an esophageal cancer cell line (Fig. 2A–C). To determine whether HMGA1 expression also plays a role in ESCC tumor growth, we constructed an HMGA1-knockdown cell line in mouse esophageal cancer AKR cells and inoculated these cells (2.5 × 10^6^) subcutaneously into C57BL/6 mice. HMGA1-knockdown cells formed tumors that are much smaller in volume and weight than those generated from control cells (*n* = 6) (Fig. 2D–F). In contrast, enforced expression of HMGA1 led to an enhanced growth of ESCC cells in the colony formation assay (Fig. 2G–I). Overexpression of HMGA1 led to much larger tumors in the syngeneic tumorigenesis assay (*n* = 6) (Fig. 2J–L). Together, our data indicate that elevated HMGA1 promotes cell proliferation and tumor growth in ESCC.

### HMGA1 upregulates pentose phosphate pathway

**Fig. 3:**
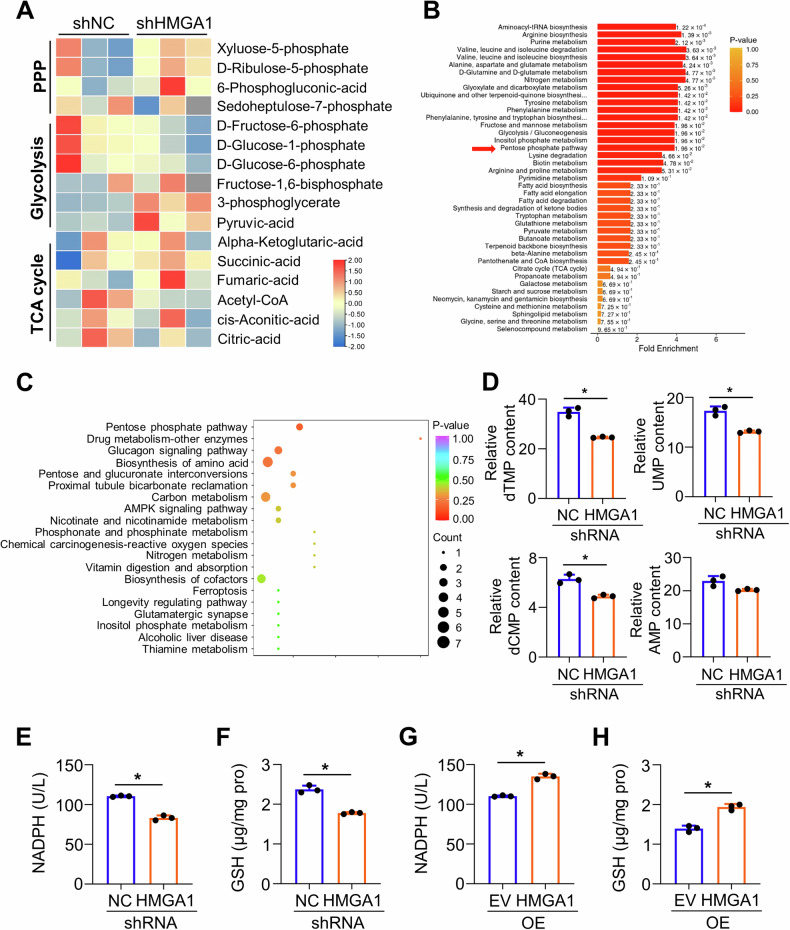
HMGA1 upregulates pentose phosphate pathway. **A**–**D** Targeted metabolomics was performed using Shimadzu LC Nexera X2 UHPLC coupled with a QTRAP 5500 LC MS/MS (AB Sciex). Chromatographic separation was performed with ACQUITY UPLC UPLC BEH Amide analytic column (Metware, Wuhan, China). **A** Heatmap of metabolites in glycolysis, PPP, TCA cycle from KYSE30-ctrl and KYSE30-shHMGA1 cells in the targeted metabolomics analysis. **B** Enrichment analysis of metabolites in KYSE30-ctrl and KYSE30-shHMGA1 cells. **C** The bubble plot of metabolite enrichment pathway. **D** Relative levels of nucleotide in KYSE30-ctrl and KYSE30-shHMGA1 cells in the targeted metabolomics analysis (*n* = 3). **E**, **F** Relative levels of NADPH (**E**) and GSH (**F**) in KYSE30 cells with HMGA1 knockdown. **G**, **H** Relative levels of NADPH (**G**) and GSH (**H**) in TE13 cells with HMGA1 overexpression. *P < 0.05, *n* = 3.

Abnormal glucose metabolism plays a critical role in tumorigenesis. To determine whether expression of HMGA1 regulates cell metabolism, we performed a targeted metabonomic analysis using control and HMGA1-knocked down KYSE30 cells. A total of 64 metabolites were detected based on LC-MS/MS detection platform and self-built database. There were 21 metabolites upregulated and 24 metabolites downregulated in HMGA1-depleted cells (Fig. 3A). KEGG enrichment analysis of the metabolomics revealed that PPP, glycolysis/gluconeogenesis, and biosynthesis of amino acids were among the mostly significantly changed pathways in HMGA1-knocked down cells (Fig. 3B, C). Further analyzing contents in the metabolomics displayed that levels of metabolites in the glycolytic pathway, such as G6P and F6P, were decreased (Fig. S2A), whereas levels of amino acids and tricarboxylic acid cycle intermediate metabolites were modestly altered in KYSE30 cells with HMGA1 deletion as compared with those in control cells (Fig. S2B, C). Consistently, nucleotides such as dTMP, UMP, and dCMP were strikingly reduced in HMGA1 deficient cells (Fig. 3D). PPP regulates de novo nucleotide biosynthesis by generating pentose phosphate, such as ribose 5-phosphate (R5P). Thus, our data indicate that HMGA1 may involve in the regulation of PPP.

NADPH and GSH, which are able to balance intracellular redox state, are important metabolites in the PPP. Thus, we examined the level of NADPH and GSH in HMGA1-manipulated cells. We found that knockdown of HMGA1 reduced (Figs. 3E, F and S2D, E) and overexpression of HMGA1 (Fig. 3G, H) increased the level of NADPH and GSH. Taken together, these findings suggest that HMGA1 upregulates the PPP in ESCC cells.

### HMGA1 upregulates the expression of TKT in ESCC

**Fig. 4:**
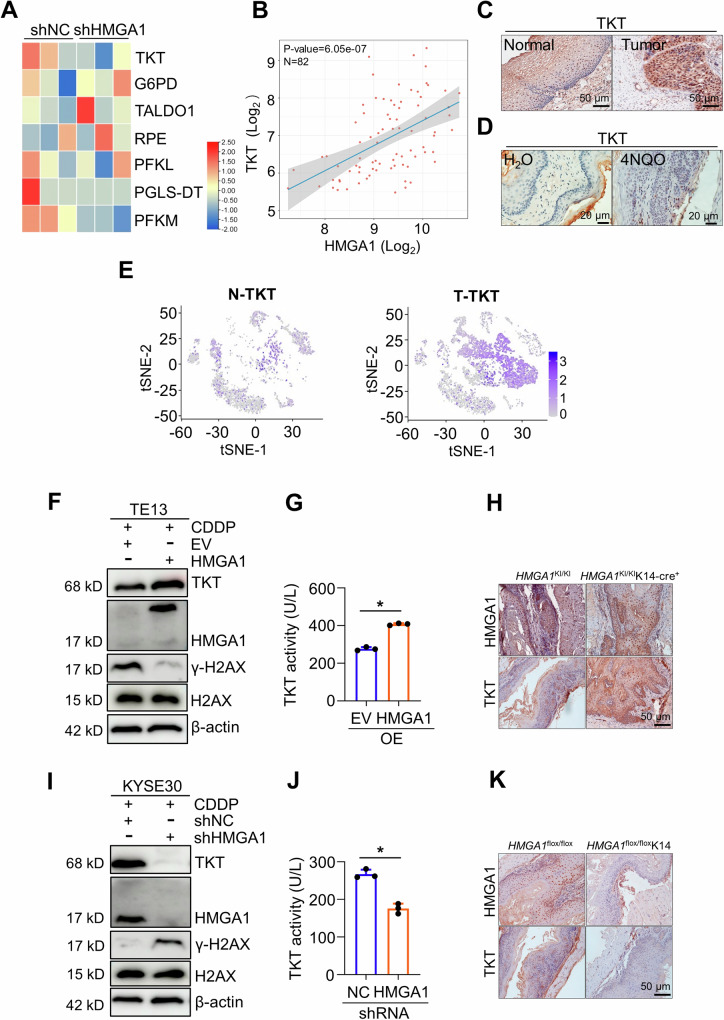
HMGA1 upregulates TKT expression. **A** RNA-seq was performed in control and HMGA1-silenced KYSE30 cells. Heatmap of partially differentially expressed genes in cell metabolism in HMGA1-silenced cells versus control cells. **B** Correlation analysis between the HMGA1 and TKT expression in ESCCs using online TCGA database. **C** Representative IHC staining of TKT in paired ESCC tissues (Scale bars: 50 μm). **D** Representative IHC staining of TKT in 4NQO-induced esophageal tumor tissue (Scale bars: 20 μm). **E** The tSNE map showed expression of TKT in ESCC tumor tissues from scRNA-seq database (GSE188900 dataset). **F**, **G** A stable cell line with HMGA1 overexpression was established in TE13 cells. Western blot (**F**) was performed to detect the expression of TKT and γ-H2AX in HMGA1-overexpressed TE13 cells exposing to CDDP (10 μM) for 16 h. The enzymatic activity (**G**) of TKT was measured in HMGA1-overexpressed TE13 cells. **H** TKT in esophageal cancers induced by 4NQO in *HMGA1*^KI/KI^ (control) and *HMGA1*^KI/KI^K14-cre^+^ mice. HMGA1 gene knock-in (*HMGA1*^KI/KI^K14-cre^+^) and its control (*HMGA1*^KI/KI^) mice were treated with 80 mg/L 4NQO in drinking water for 5 months for the induction of esophageal cancer (*n* = 6). Representative images of esophageal sections stained with HMGA1 and TKT antibody are shown. Scale bars, 50 μm. **I**, **J** A stable cell line with HMGA1-knocked down was established in KYSE30 cells. Western blot (**I**) was performed to detect the expression of TKT and γ-H2AX in HMGA1-knocked down KYSE30 cells exposed to CDDP (10 μM) for 16 h. The enzymatic activity (**J**) of TKT was measured in HMGA1-knocked down KYSE30 cells. **K** TKT in esophageal cancers induced by 4NQO in *HMGA1*^flox/flox^ and *HMGA1*^flox/flox^K14 mice. Conditional *HMGA1* gene knock-out in esophagus (*HMGA1*^flox/flox^K14) and its control (*HMGA1*^flox/flox^) mice were treated with 80 mg/L 4NQO in drinking water for 5 months for the induction of esophageal cancer (*n* = 6). Representative images of esophageal sections stained with HMGA1 and TKT antibody are shown. Scale bars, 50 μm. Student’s *t* test was used for the statistical analysis. *P < 0.05, *n* = 3.

To explore how HMGA1 affects glucose metabolism, such as PPP, we conducted a transcriptome sequencing in KYSE30 cells with or without HMGA1 knockdown. Multiple genes encoding enzymes in glucose metabolism were reduced in HMGA1-depleted cells. Some of the differentially expressed genes (DEGs) were presented in the heatmap (Fig. 4A). Interestingly, transketolase (TKT), a key enzyme in the non-oxidative PPP, was strikingly downregulated due to the silencing of HMGA1 in KYSE30 cells (Fig. 4A). TKT catalyzes the reversible reaction in non-oxidative PPP that exchanges metabolites with the glycolytic pathway. It induces DNA synthesis and cell cycle progression in the form of R5P accumulation [37]. We analyzed the correlation between HMGA1 and TKT in ESCC in TCGA database and found that there was a positive correlation between HMGA1 and TKT (Fig. 4B), consistent with the results from RNA sequencing (Fig. 4A). Results from IHC also showed that TKT was more strongly expressed in esophageal carcinomas than that in adjacent normal esophageal epithelial tissues both in human (Fig. 4C) and in mice (Fig. 4D). TKT was highly expressed both in the nucleus and in the cytoplasm (Fig. 4C, D), sharing a similar pattern with HMGA1 staining (Fig. 1F, H). Single-cell RNA sequencing results also showed that TKT was highly expressed in epithelial tumor tissues of ESCC (Figs. 4E and S3A, B), whereas G6PD, a rate-limiting enzyme in the oxidative PPP, was only weakly expressed in esophageal tumor tissues (Fig. S3C).

To elucidate the role of HMGA1 in the regulation of TKT, we modified the expression of HMGA1 and determined the level and activity of TKT. Enforced expression of HMGA1 markedly increased the level and enzymatic activity of TKT, but not that of G6PD in the oxidative PPP (Figs. 4F, G and S3D–F). TKT participates in PPP and regulates de novo nucleotide biosynthesis. When nucleotide biosynthesis is reduced, DNA damage repair is delayed, and γ-H2AX, a marker for DNA damage, is continuously expressed. Cisplatin (CDDP), a DNA damage drug, effectively induces DNA damage, disrupting DNA replication and transcription. Consistently, under the treatment of CDDP, cells bore a high level of γ-H2AX (Fig. 4F). Enforced expression of HMGA1 reduced CDDP-induced γ-H2AX (Fig. 4F). To further characterize the impact of HMGA1 on DNA damage response (DDR), we detected the expression of γ-H2AX in HMGA1-manipulated cells treated with different dosages or exposure time of CDDP. It showed that CDDP treatment led to a dosage- and time-dependent increase of γ-H2AX in parental cells (Fig. S4). Overexpression of HMGA1 mitigated CDDP-induced cumulation of γ-H2AX in TE13 cells in a dosage- and time-dependent manner (Fig. S4A, B). To further validate the regulatory role of HMGA1 in the expression of TKT, we detected TKT in esophageal cancer in *HMGA1* conditional knock-in (*HMGA1*^KI/KI^K14-cre^+^) mice. We induced esophageal cancer with 5-month-treatment of 4NQO in *HMGA1*^KI/KI^K14-cre^+^ mice. Esophageal cancer tissues in HMGA1 conditional knock-in (*HMGA1*^KI/KI^K14-cre^+^) mice displayed a much higher proportion of TKT-positive cells as compared with those in esophageal cancers induced in control mice (*HMGA1*^KI/KI^) (Fig. 4H).

To further validate the role of HMGA1 in the regulation of TKT and its resultant DNA repair, we performed similar experiments in HMGA1 knockdown cells. Depletion of HMGA1 downregulated the level and enzymatic activity of TKT, but did not affect the enzyme activity of G6PD (Figs. 4I, J and S3G–J). Knockdown of HMGA1 enhanced the accumulation of γ-H2AX in KYSE30 cells (Figs. 4I and S4C, D). *HMGA1*-conditional knock out (*HMGA1*^flox/flox^K14-cre^+^) mice bore fewer TKT-positive cells in the esophageal tissues (Fig. 4K). Together, our data suggest that HMGA1 upregulates TKT expression. DNA damage is ameliorated following expression of HMGA1, which may be credited to HMGA1-mediated upregulation of TKT and resultant nucleotide biosynthesis.

### HMGA1 activates PPP by upregulating TKT

**Fig. 5:**
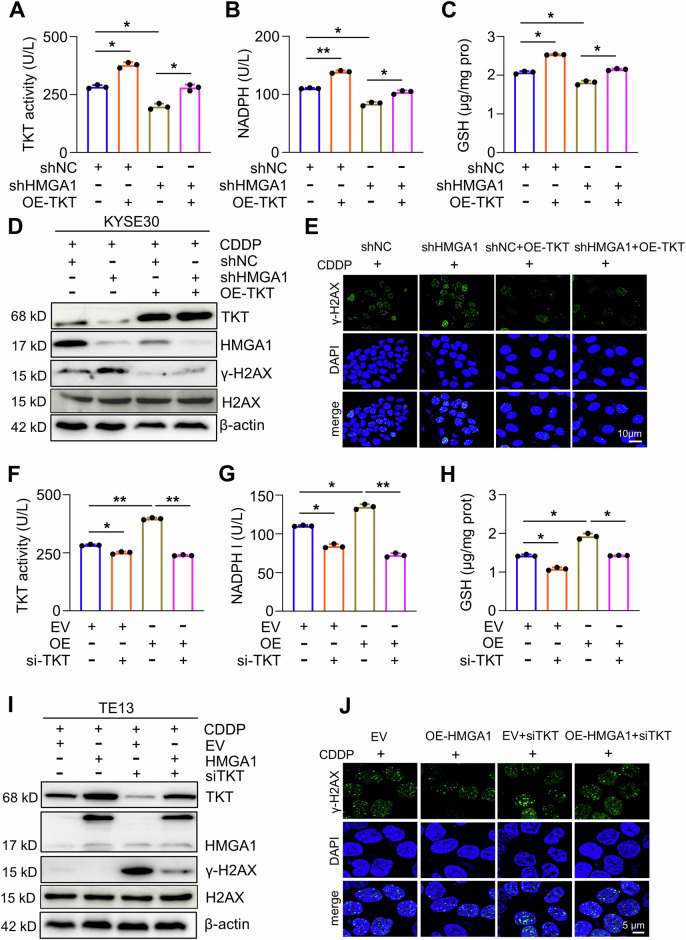
TKT mediates HMGA1-upregulated PPP. **A**–**C** Control and HMGA1-knocked down KYSE30 cells were transfected with Flag-tagged TKT. TKT enzyme activity (**A**), NADPH (**B**), and GSH (**C**) were determined in cells. **D**, **E** Enforced expression of TKT ameliorates HMGA1 depletion-induced DNA damage. Control and HMGA1-knocked down KYSE30 cells were transfected with pcDNA3.1/TKT for 24 h. Cells were then treated with 10 μm CDDP for another 16 h. After the treatment, cells were collected for determining the expression of γ-H2AX (Ser139), HMGA1, and TKT by western blotting (**D**) or subjected to the immunofluorescence staining for detecting the expression of γ-H2AX (Ser139) (**E**). Scale bar, 10 μm. **F**–**H** Control and HMGA1-overexpressed TE13 cells were transfected with TKT siRNA and detected for TKT enzyme activity (**F**), NADPH (**G**), and GSH (**H**). **I**, **J** Depletion of TKT reverses HMGA1-alleviated DNA damage. Control and HMGA1-overexpressed TE13 cells were transfected with TKT siRNA for 24 h. Cells were then treated with 10 μm CDDP for another 16 h. After the treatment, cells were used for determining the expression of γ-H2AX (Ser139), HMGA1, and TKT by western blotting (**I**) or fixed for the immunofluorescence detection of γ-H2AX (Ser139) (**J**). Scale bar, 5 μm.

To determine the role of TKT in the upregulation of PPP by HMGA1, we conducted a series of rescue experiments. Overexpression of TKT ameliorated HMGA1 depletion-induced exhaustion of NADPH, GSH, and TKT activation (Figs. 5A–C and S5A–C). We previously showed that HMGA1 knockdown resulted in a decrease in nucleotides (Fig. 3D) and an increase in DNA damage (Fig. 4I). TKT overexpression activates the non-oxidative PPP and promotes DNA damage repair by elevating the synthesis of nucleotides [19]. Thus, we performed a rescue assay to detect the expression of γ-H2AX in HMGA1-modified cells with TKT manipulations. HMGA1 knockdown led to a substantial increase of γ-H2AX, which was mitigated by the overexpression of TKT (Figs. 5D, E and S5D, E). Enforced expression of TKT ameliorated HMGA1 depletion-aggravated DNA damage and reduced γ-H2AX in the western blot detection (Figs. 5D and S5D) and in the immunofluorescence analysis (Figs. 5E and S5E). In a stark contrast, knockdown of TKT abrogated HMGA1-induced elevation of NADPH and GSH and activation of TKT (Fig. 5F–H). Under the treatment of CDDP for 16 h, the level of γ-H2AX was markedly reduced after HMGA1 overexpression, which was reversed by TKT silence (Figs. 5I and S5F). Furthermore, we performed an immunofluorescence staining with antibody against γ-H2AX to label the damaged DNA and found that silencing TKT abrogated the protection of HMGA1 overexpression on DNA damage and markedly increased the staining of γ-H2AX (Figs. 5J and S5G). Together, these results support our notion that TKT mediates HMGA1-activated PPP and DNA damage repair.

### HMGA1 promotes TKT transcription

**Fig. 6:**
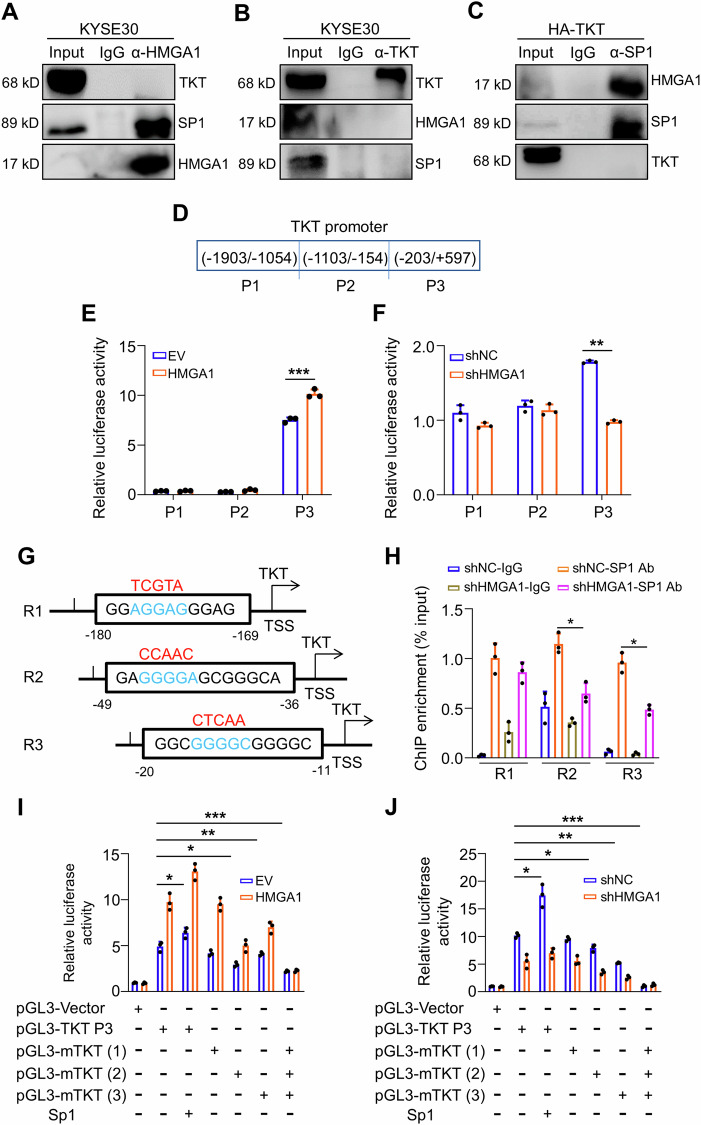
HMGA1 promotes the transcription of TKT by interacting with SP1. **A** Co-immunoprecipitation (co-IP) assay was used for the detection of interactions among endogenous HMGA1, TKT, and SP1 in KYSE30 cells. Antibody against HMGA1 was used for the pulldown. **B** No direct interaction exerts between TKT and HMGA1 or Sp1. A specific antibody against TKT was used for immunoprecipitating TKT and its complex from TKT-overexpressed KYSE30 cells. TKT, HMGA1, and SP1 were detected in the pulled-down complex. **C** Sp1 coimmunoprecipitates with HMGA1, but not TKT. A specific antibody against Sp1 was used for immunoprecipitating Sp1 and its complex from TKT-overexpressed KYSE30 cells. SP1, HMGA1, and TKT were detected in the pulled-down complex. **D** Schematic of the truncated TKT promoter sequences. **E** Transcriptional activity of truncated TKT promoter fragments was measured by luciferase reporter assays in 293T cells stably transfected with HMGA1 overexpression plasmid. **F** Transcriptional activity of truncated TKT promoter fragments was measured by luciferase reporter assays in KYSE30 cells stably transfected with HMGA1 shRNA plasmids. **G** The potential AT-hook sequences for HMGA1 and/or Sp1 binding were mutated as indicated. **H** ChIP assays were performed to study the binding of Sp1 to the TKT promoter region (−187/−54, −173/−25, −27/100) in KYSE30 cells with or without HMGA1 knockdown. **I**, **J** Transcriptional activity of the wild-type or mutated TKT promoter sequences was measured by luciferase reporter assays in 293T cells (**I**) and KYSE30 cells (**J**) with HMGA1 manipulations. Student’s *t* test was used for the statistical analysis. *P < 0.05, **P < 0.01, and ***P < 0.001, *n* = 3.

HMGA1 is a chromatin remodeling factor, which influences the binding of transcriptional factors to their target gene promoters to regulate downstream gene expression although HMGA1 itself is not a transcriptional factor. We identified that HMGA1 upregulated TKT mRNA (Fig. S3D, E). Thus, we postulated that HMGA1 possibly regulates TKT at the transcriptional level. We screened the top transcriptional factors that bind to the TKT promoter area through the Genebank database and identified that specificity protein 1 (Sp1) was the first hit. We verified the interaction between Sp1 and HMGA1 (Fig. 6A–C), but not between Sp1 and TKT, or TKT and HMGA1, with the co-IP assays (Fig. 6A–C). We searched the 2.5 kb region around the TKT transcription start site (TSS) for putative Sp1/HMGA1-binding sites. To determine which region(s) of TKT promoter is targeted by HMGA1 and hence plays a role in HMGA1-mediated regulation of TKT, sequences including different areas of TKT promoter were cloned and examined for identifying the elements responsive to Sp1/HMGA1 in ESCC cells (Fig. 6D). TKT promoter region was divided into 3 segments (P1-3) and cloned into a luciferase vector (Fig. 6D). We found that luciferase activity of P3 (−203 to +597) promoter area was substantially increased with HMGA1 overexpression and decreased with HMGA1 knockdown, whereas the promoter area P1 and P2 were irresponsive to HMGA1 manipulations (Fig. 6E, F).

Around P3 (−203 to +597) area, three AT-rich sequences predicted to be bound by Sp1 were identified (Fig. 6G). We defined these three sites as R1 (−180 to −169), R2 (−49 to −36), and R3 (−20 to −11) (Fig. 6G). ChIP assay confirmed that Sp1 bound to all 3 sites in this area (Fig. 6H). However, only binding to R2 (−49 to −36) and R3 (−20 to −11) by Sp1 were substantially reduced due to the depletion of HMGA1 (Fig. 6H). To further validate the impact of HMGA1 on the binding of Sp1 to TKT promoter, we constructed mutants with mutations in these three Sp1-binding sites (Fig. 6G, sequences labeled in red). Consistently, the luciferase reporter activity of TKT promoter with mutated Region 2 and 3 sites was substantially reduced in spite of the overexpression of HMGA1 (Fig. 6I), supporting the notion that HMGA1 affects the binding of Sp1 to the promoter of TKT at R2 (−49 to −36) and R3 (−20 to −11). Mutation in R1 (−180 to −169) was unable to affect the promotion of HMGA1 on TKT promoter activity (Fig. 6I). In addition, depletion of HMGA1 markedly abrogated the transcriptional activity of wild-type pGL3-TKT P3 (including R1-3) and mutated pGL3-mTKT R1, but showed only a marginal impact on the luciferase activity of mutated pGL3-mTKT R2 and R3 (Fig. 6J). Taken together, our data suggest that HMGA1 assists Sp1 to bind to TKT promoter and promotes its transcription.

### Inhibition of TKT suppresses the oncogenic activity of HMGA1

**Fig. 7:**
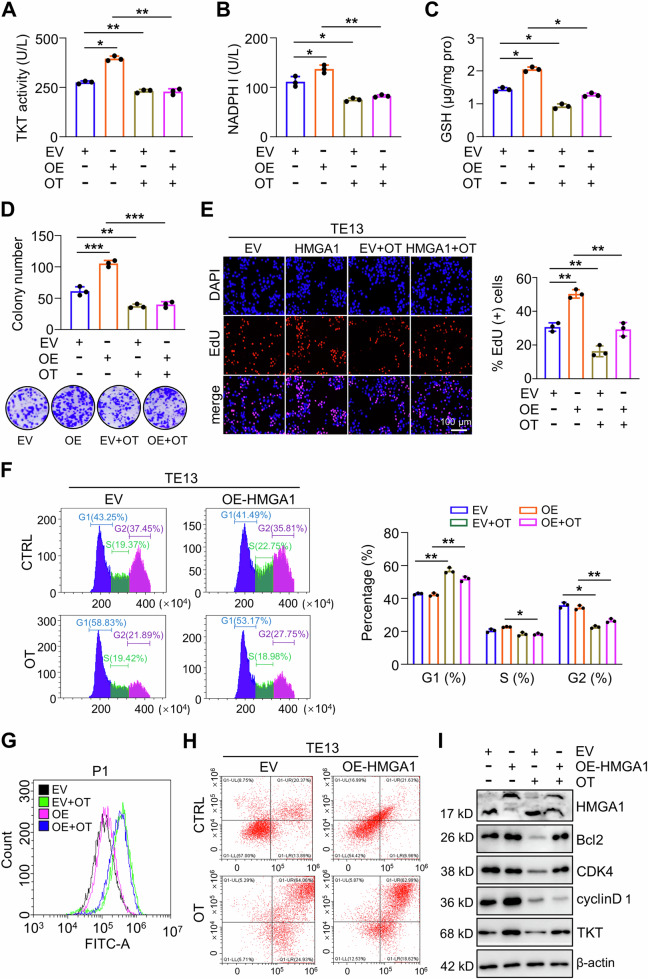
Inhibition of TKT suppresses the oncogenic activity of HMGA1. **A**–**E** Control and HMGA1-overexpressed TE13 cells were treated with OT (5 mM) for 24 h. **A** TKT enzyme activity; **B** NADPH; **C** GSH; **D** Colony formation of the cells was determined. **E** Cell proliferation was evaluated by EdU incorporation assay in the cells. Scale bar: 100 μm. **F** Flow cytometry was performed to analyze cell cycle distribution of TE13 cells with or without HMGA1 overexpression cultured in the presence or absence of 5 mM OT for 24 h (*n* = 3). **G** ROS level was measured in TE13 cells with or without HMGA1 overexpression treated with 5 mM OT for 8 h. ROS were determined by DCF staining and analyzed with an ROS Assay Kit from Beyotime Biotechnology. **H** Flow cytometry was performed to analyze cell apoptosis of TE13 cells treated with or without 10 mM OT for 72 h (*n* = 3). **I** Western blot analyses were performed for the detection of cell cycle and apoptotic proteins in OT-treated TE13 cells with or without HMGA1 overexpression.

To evaluate the importance of TKT-mediated PPP in the maintenance of oncogenic phenotype of HMGA1, we tested the effect of TKT inhibitor oxythiamine (OT) on the function of HMGA1 in ESCC cells. As expected, administration of OT significantly reduced the enzymatic activity of TKT and decreased the levels of NADPH and GSH in TE13 cells regardless of the presence of HMGA1 overexpression (Fig. 7A–C). We next tested the colony formation in TE13 cells with HMGA1 overexpression under the treatment of OT. Enforced expression of HMGA1 enhanced TE13 cell colony formation, which was strikingly reduced with the treatment of OT (Fig. 7D). EdU assay further confirmed that inhibition of TKT with OT reduced HMGA1-promoted cell growth (Fig. 7E). It was reported that HMGA1 suppression inhibits tumor progression by blocking cell cycle [38]. Flow cytometry assay showed that transduction of HMGA1 led to statistically more cells in S phase (Fig. 7F). After treatment with OT, cells in G1 phase increased and those in S and G2 phases decreased markedly in spite of the existence of HMGA1 overexpression (Fig. 7F). In order to clarify the effect of TKT inhibition on the redox status of HMGA1-overexpressed cells, ROS level in cytoplasm was measured after TKT inhibition. The results showed that the ROS level of cells treated with OT was significantly increased (Fig. 7G). There was no significant difference between control and HMGA1-overexpressed cells in the presence of OT (Fig. 7G). In addition, there were more cells committed to apoptosis after OT treatment than those without OT treatment (Fig. 7H). Consistently, HMGA1-induced upregulations of cell cycle regulators cyclin D1 and CDK4 and anti-apoptotic protein Bcl2 were decreased due to the treatment of OT (Fig. 7I). Taken together, our results demonstrate that HMGA1 promotes the malignant phenotype of ESCC cells, which could be blocked with the inhibition of TKT.

### Suppression of TKT abrogates HMGA1-induced tumor growth

**Fig. 8:**
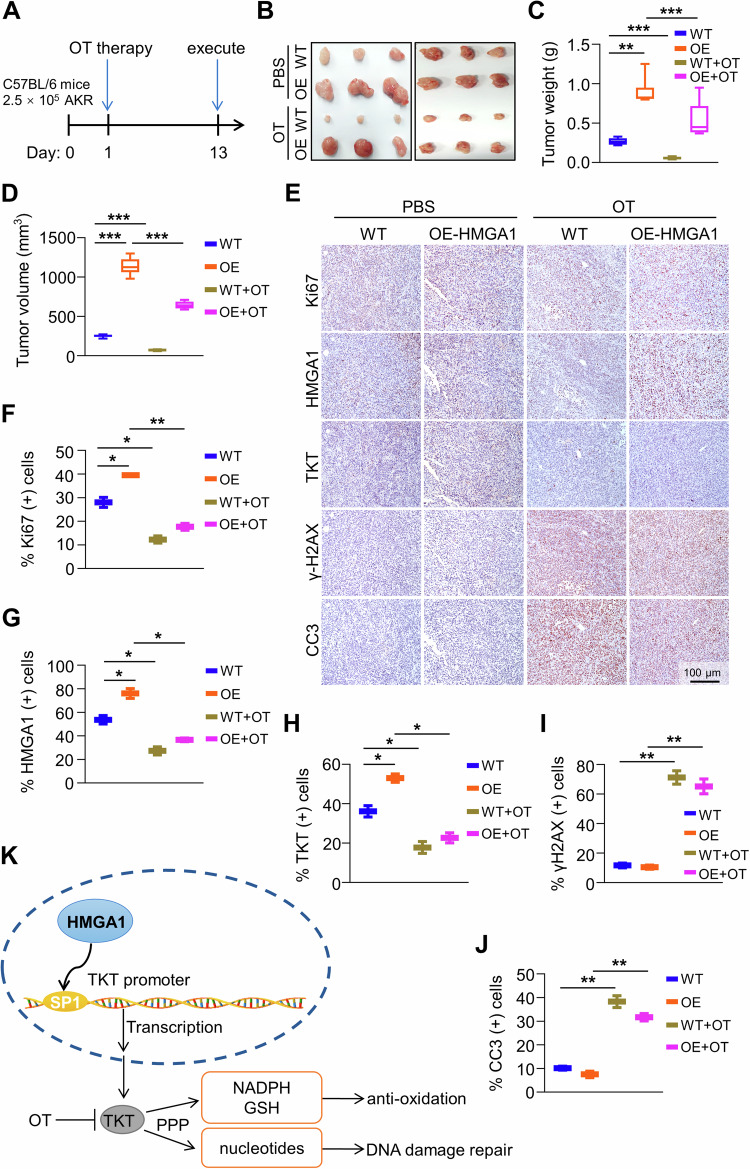
Suppression of TKT abrogates HMGA1-induced ESCC tumor growth. AKR-WT and AKR-HMGA1 overexpression cells (2.5 × 10^5^/mouse) were injected into flanks of C57BL/6 mice by s.c. The second day after seeding the cells, TKT inhibitor OT (300 mg/kg/day) was applied to the mice by gavage. Tumor volumes were monitored for 13 d. **A** Timeline for the establishment of the syngeneic mouse model and the treatment of OT. **B** Representative images of tumors formed in C57BL/6 mice (*n* = 6). **C** Tumor weights, and **D** tumor volume in mice treated with or without OT. **E**–**J** Immunohistochemical staining and quantitation of paraffin-embedded tissue sections for HMGA1, TKT, cleaved-caspase 3, γ-H2AX, and Ki-67 in tumors from mice treated with or without OT (*n* = 6). Scale bar, 100 μm. Data are presented as the means ± S.D., and significant differences are indicated as *P < 0.05, **P < 0.01, and ***P < 0.001. **K** Schematic diagram depicting that HMGA1 promotes ESCC progression by elevating TKT-mediated upregulation of PPP.

In order to study the effect of TKT inhibition on HMGA1-induced tumor progression, we applied TKT inhibitor OT or PBS into mice by gavage every day. AKR cells transfected with empty vector or HMGA1 construct were inoculated subcutaneously into C57BL/6 mice. From the second day, the mice were given 300 mg/kg/day OT for 12 days (Fig. 8A). The tumor weight and volume in mice treated with OT were significantly smaller than those in mice treated with PBS (Fig. 8B–D). The percentage of Ki67, HMGA1, and TKT-positive cells was higher in tumors generated from HMGA1-transduced cells than that in tumors generated from vector-transduced cells (Fig. 8E–H). Expression of γ-H2AX and cleaved-caspase 3 was low in HMGA1-transduced tumors (Fig. 8E, I, J). Administration of OT led to a strikingly reduction in cells with positive Ki67 and an increase in the expression of γ-H2AX and cleaved-caspase 3 (Fig. 8E–J). These data indicate that HMGA1 promotes tumor progression. Targeting TKT blocks HMGA1-induced tumor growth. TKT inhibitor could be a potential therapeutic agent for ESCCs with high expression of HMGA1 (Fig. 8K).

## Discussion

HMGA1 is a member of the HMGA family, which exerts its biological functions as a structural transcriptional factor. HMGA1 is positively correlated with the degree of cancer progression and an unfavourable marker for clinical prognosis. HMGA1 has also recently been characterized as a key regulator for glucose homeostasis [39]. HMGA1 promotes the transcriptional regulation of genes related to glucose homeostasis [40, 41].

In the current study, we demonstrated that HMGA1 was highly expressed in ESCCs. Upregulation of TKT by HMGA1 elevated the enzyme activity of TKT and hence activated non-oxidative PPP and intensified the malignant behaviour of ESCC. The findings showed a metabolic mechanism for the role of HMGA1 in promoting ESCC tumorigenesis and development. Our metabolomics results showed a decrease in multiple metabolites of the PPP due to the knockdown of HMGA1. Because of the bidirectional regulation of TKT in the conversion between glycolysis and PPP, metabolites in the glycolytic pathway, such as G6P and F6P, were also deregulated by HMGA1/TKT.

We found that HMGA1 knockdown led to an increased DNA damage. Repair of DNA damage needs sufficient nucleotides for the synthesis of DNA strand. Our metabolomics assay revealed a substantial reduction in nucleotides (dTMP, dCMP, and UMP) in HMGA1 knockdown cells. Surprisingly, R5P and Xu5P increased in HMGA1 knockdown cells, this may be due to the increased DNA damage caused by HMGA1 knockdown, and the cells accelerated the production of R5P and Xu5P in order to repair the DNA damage, forming a feedback regulatory loop. We found that suppression or knock-down of TKT markedly inhibited cell and tumor growth (Figs. 7D, E, and 8B), regardless of the accumulation of ribose intermediates, indicating that ribose is not a restrictive factor in cancer growth. Although, based on our current data, we are unable to determine the accumulation of riboses in TKT knockdown cells is the result of reduced consumption or increased yield of riboses, it is indeed a meaningful question that needs to be addressed in the future.

PPP meets the needs of organisms to use NADPH in reductive biosynthesis [42]. TKT is an enzyme in the non-oxidative branch of PPP and catalyzes the reversable metabolism between glycolysis and PPP to provide phosphate sugar for main carbohydrate metabolism pathways [23]. On the other hand, as a key enzyme in the oxidative PPP pathway, G6PD catalyzes the oxidation of G6P to 6-phosphate gluconate, which finally contributes to the production of NADPH and hence maintains normal redox potential in cells [43]. Researches showed that expression of G6PD in tumor cells is higher than that in normal cells [44, 45]. In our experimental setting, however, we observed no changes in enzyme activity of G6PD following the manipulations of HMGA1 in ESCC cells. On the contrary, TKT knockdown resulted in a decrease of NADPH and GSH and an increase of ROS. Of note, one question that remains unanswered is why the level of NADPH, a metabolite from the oxidative arm, decreased in HMGA1 depletion cells. One possible explanation is that knocking down TKT leads to a gradual accumulation of R5P and Ru5P that eventually blocks the oxidation arm of PPP. In the future, application of [1-^2^H]-glucose to trace NADPH production in the TKT knockdown cells should be able to solve the puzzle.

Thiamine, also known as vitamin B1, is one of the key vitamins necessary for maintaining the function of a cell and an organism. OT is an antivitamin of thiamine. It interferes with the enzyme activity of thiamine and inhibits the conversion of glucose into pentose phosphate by suppressing TKT. In our study, we investigated the effect of OT on tumor suppression by interfering the PPP. Due to the high tolerance of OT by human healthy cells, OT was used in the treatment of cancers and infections [46, 47]. In order to make a direct comparison of the effects of OT on cancer cells (HeLa) and fibroblasts, Malinowska et al. exposed these cells to OT and determined the values of GI50 (the concentration of OT that reduces total cell growth by 50%), IC50 (half-maximal inhibitory concentration), and SI (selectivity index showing the ratio of IC50 for OT concerning fibroblasts versus cancer cells). The authors found that OT shows a much stronger cytostatic effect on tumor cells as compared to normal human fibroblasts with an SI being 150 (IC50 of OT for fibroblasts is 150 fold higher than that in cancer cells) [47]. Thus, the side effect of OT is minimal and off-target effects of the compound may not be a concern in the treatment of cancers.

Mounting evidence demonstrates that the link between cancer metabolism and DNA damage response (DDR) can be exploited therapeutically [48–50]. In the current study, we found that HMGA1 overexpression reduced CDDP-induced DNA damage (Fig. 4F), whereas HMGA1 or TKT knockdown would aggravate DNA damage (Fig. 5D, I). Through the rescue experiments, we demonstrated that TKT overexpression reduced DNA damage in HMGA1 knockdown cells. Activation of TKT increased the generation of nucleotides, which provide substrates for DNA damage repair, supporting our notion that TKT mediates the function of HMGA1 in DNA damage repair. Thus, our findings provide a rationale for targeting TKT in the therapy of tumors with upregulated HMGA1.

Although there is growing evidence showing that TKT promotes the progression of various cancers, the specific biological mechanisms underlying the function of TKT remain incompletely understood. Our study demonstrated that HMGA1 primarily upregulated the transcription of TKT. HMGA1 interacted with the transcriptional factor Sp1 to activate its transcriptional activity. Epigenetic modifications, such as DNA methylation, histone methylation, acetylation, and chromatin remodeling, have been shown to have a considerable impact on gene expression. In the future, it is interesting to explore the underlying mechanisms by which HMGA1 affects TKT expression in these aspects.

To summarize, the current study demonstrated that HMGA1 was increased in ESCCs. Downregulation of HMGA1 inactivated the PPP by inhibiting TKT transcription. HMGA1 bound to Sp1 in TKT promoter and enhanced the transcription of the later, and hence promoted non-oxidative PPP and the proliferation of ESCC cells. We further demonstrated that targeting TKT suppressed the growth of ESCC tumors. Our study shed new light in the development of novel therapeutic strategy for ESCC by suppressing the PPP.

### Supplementary information

Supplemental Material

Original Data

## Data Availability

The authors declare that all the data supporting our findings in the study are available within the paper.
