# Supplementary material for: HMGA1 promotes the progression of esophageal squamous cell carcinoma by elevating TKT-mediated upregulation of pentose phosphate pathway
Source: Cell Death Dis. 2024 Jul 30;15(7):541. doi: 10.1038/s41419-024-06933-x (PMC11289123; doi:10.1038/s41419-024-06933-x)
Supplement: Supplementary file 1 — Supplemental Material [file 41419_2024_6933_MOESM1_ESM.pdf]

Supplementary Table S1

Gene/plasmids	Primers	Primer sequences
HMGA1-human	forward primer	CGAGAAAAGGACGGCACTGA
	reverse primer	TTGTGGTGGTTTTCCGGGTC
TKT-human	forward primer	GACAACCTTGTGGCCATTCT
	reverse primer	TCTGCTCAGCCATGTTTTTG
GAPDH-human	forward primer	AACGGATTTGGTCGTATTGG
	reverse primer	TTGATTTTGGAGGGATCTCG
HMGA1-shRNA		GAAGTGCCAACACCTAAGAGA
TKT-pcDNA 3.1	forward primer	GTGGTGGAATTCATGGAGAGCTACCACA AGCCTGACCAG
	reverse primer	TCTAGACTCGAGCTAGGCCTTGGTGATG AGGCCCCTCACA
HA-Flag-TKT	forward primer	GGACCGGTTCTAGAGCCACCATGGAGAG CTACCACAAGCC
	reverse primer	TCTGGAACATCGTATGGGTAGGCCTTGGT GATGAGGCCCC
HA-Flag-HMGA1	forward primer	GGTTCTAGAGCCACCATGAGTGAGTCGA GCTCGAA
	reverse primer	AGTCCTCGGAGGAGGAGCAGTACCCATA CGATGTTCCAGA
HA-Flag-Sp1	forward primer	GGACCGGTTCTAGAGCCACCATGAGCGA CCAAGATCACTC

	reverse primer	TCTGGAACATCGTATGGGTAGAAGCCATT GCCACTGATAT
siRNA-TKT	forward primer	CCGGCAAAUACUUCGACAATTA
	reverse primer	UUGUCGAAGUAUUUGCCGGTT
TKT promoter-P1	forward primer	GGTACCGAGCTCTTTTTCCATTACTCCTA GGTCCCCAGA
	reverse primer	ACTTAGATCGCAGATTACAAATTATTAGG GCCTAGAAACA
TKT promoter-P2	forward primer	GGTACCGAGCTCTTCAATAATCTCCCTAA AAGTCAGTAG
	reverse primer	ACTTAGATCGCAGATTCGAAAGCCTCTCA GTCTCCCTCCT
TKT promoter-P3	forward primer	GGTACCGAGCTCTTGACAAGTCCACGGA GGACTGTGTGG
	reverse primer	ACTTAGATCGCAGAT CGCGAGCCCATCCCCCGCGCCACCA
ChIP-R1	forward primer	ACTGTGTGGAGGAGGGAGAC
	reverse primer	AGGCGCTGGGCCGCTGCGGA
ChIP-R2	forward primer	GGAGACTGAGAGGCTTTCGA
	reverse primer	GGGCCGCCTCCTGCCCGCTC
ChIP-R3	forward primer	CCCCAGGCGGGGCGGGGCTG
	reverse primer	AGGCCGGGCGCGGGGCGGGG

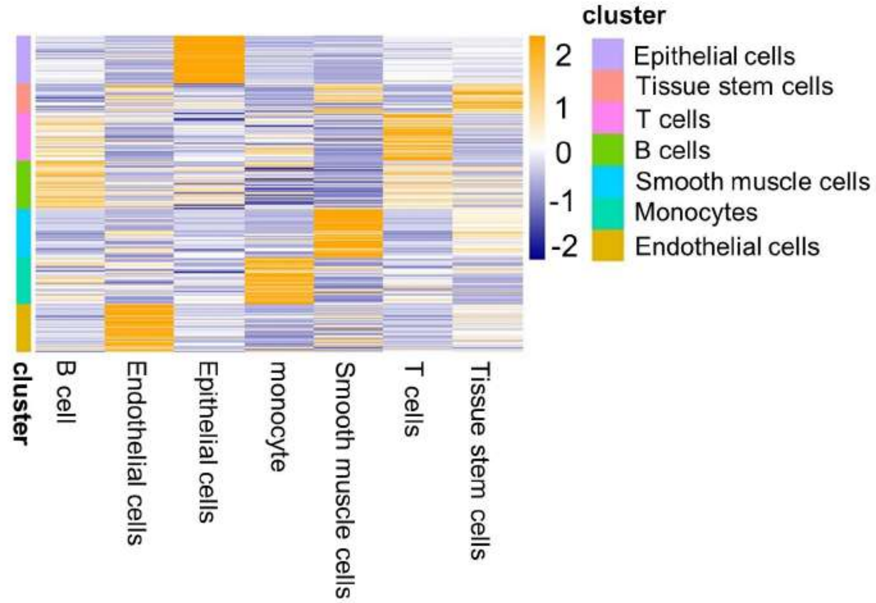

cluster
Epithelial cells
Tissue stem cells
T cells
B cells
Smooth muscle cells
Monocytes
Endothelial cells
2
1
0
-1
-2
cluster
B cell
Endothelial cells
Epithelial cells
monocyte
Smooth muscle cells
T cells
Tissue stem cells

Supplementary Fig. 1 The tSNE map of the single cell analysis in ESCCs and the scape was colored by cell subtypes and shown in Fig. 1A. The heatmap showed the expression levels of different cell subtype signatures in scRNA-seq data from the GEO database (GSE188900 dataset).

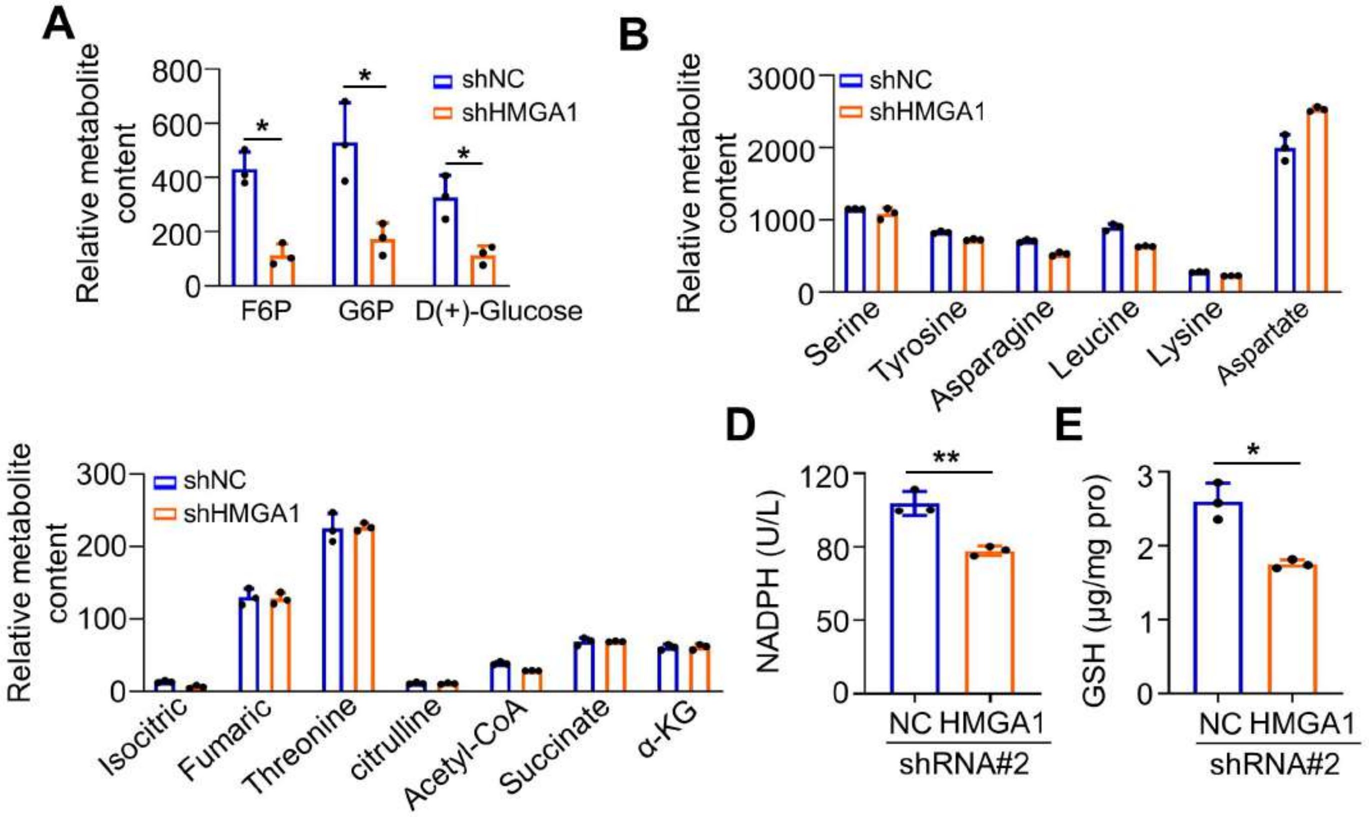
A
Relative metabolite content
800
600
400
200
0
*
*
*
shNC
shHMGA1
F6P
G6P
D(+)-Glucose
B
Relative metabolite content
3000
2000
1000
0
shNC
shHMGA1
Serine
Tyrosine
Asparagine
Leucine
Lysine
Aspartate
C
Relative metabolite content
300
200
100
0
shNC
shHMGA1
Isocitric
Fumaric
Threonine
citrulline
Acetyl-CoA
Succinate
α-KG
D
NADPH (U/L)
120
80
50
0
**
NC HMGA1
shRNA#2
E
GSH (μg/mg pro)
3
2
1
0
*
NC HMGA1
shRNA#2

Supplementary Fig. 2 Levels of glycolytic and amino acid metabolites in the targeted metabolomics analysis of HMGA1 intact and deficient KYSE30 cells. A. Relative levels of glycolytic metabolites in KYSE30-ctrl and KYSE30-shHMGA1 cells (n = 3). **B**, **C.** Relative levels of amino acids and tricarboxylic acid cycle intermediate metabolite in KYSE30-ctrl and KYSE30-shHMGA1 cells (n = 3). **D, E.** Relative levels of NADPH and GSH in KYSE30 cells with HMGA1 knockdown. Data are presented as the means ± S.D., and significant differences are indicated as * $P < 0.05$, ** $P < 0.01$.

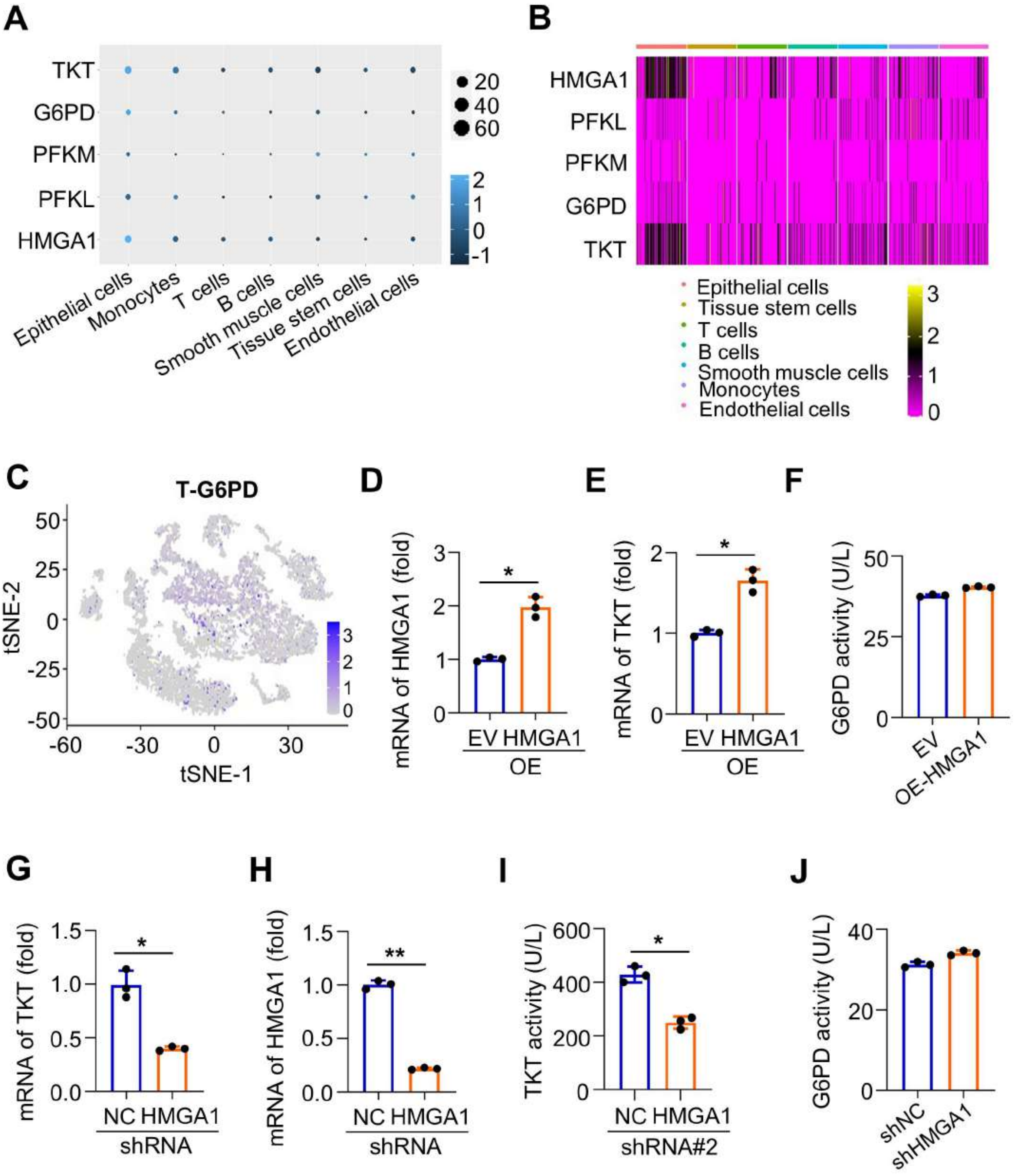

A
TKT
G6PD
PFKM
PFKL
HMGA1
20
40
60
2
1
0
-1
Epithelial cells
Monocytes
T cells
B cells
Smooth muscle cells
Tissue stem cells
Endothelial cells
B
HMGA1
PFKL
PFKM
G6PD
TKT
Epithelial cells
Tissue stem cells
T cells
B cells
Smooth muscle cells
Monocytes
Endothelial cells
3
2
1
0
C
T-G6PD
tSNE-2
tSNE-1
D
mRNA of HMGA1 (fold)
EV HMGA1
OE
E
mRNA of TKT (fold)
EV HMGA1
OE
F
G6PD activity (U/L)
EV
OE-HMGA1
G
mRNA of TKT (fold)
NC HMGA1
shRNA
H
mRNA of HMGA1 (fold)
NC HMGA1
shRNA
I
TKT activity (U/L)
NC HMGA1
shRNA#2
J
G6PD activity (U/L)
shNC
shHMGA1

Supplementary Fig. 3 Metabolic enzymes in HMGA1-manipulated ESCC cells. A. The dot plot map showed the expression of HMGA1 and different metabolic enzyme genes in a variety of cell subtypes in ESCCs from scRNA-seq data in the GEO database (GSE188900 dataset). **B**. The tSNE map showed expression levels of HMGA1 and various metabolic enzyme genes in different cell subtypes in ESCCs. **C**. The tSNE map showed expression of G6PD in ESCCs from scRNA-seq data in the GEO database (GSE188900 dataset). **D, E.** Levels of HMGA1 (**D**) and TKT (**E**) mRNA were determined by RT-qPCR in HMGA1-overexpressed TE13 cells. Samples were normalized to GAPDH mRNA. **F.** The enzymatic activity of G6PD was measured in HMGA1-overexpressed TE13 cells. **G, H.** Levels of HMGA1 (**G**) and TKT (**H**) mRNA were determined by RT-qPCR in HMGA1-knocked down KYSE30 cells. Samples were normalized to GAPDH mRNA. **I.** The enzymatic activity of TKT was measured in HMGA1-knocked down KYSE30 cells. **J**. The enzymatic activity of G6PD was measured in HMGA1-knocked down KYSE30 cells. Data are presented as the means ± S.D., and significant differences are indicated as * $P < 0.05$, ** $P < 0.01$. $n = 3$.

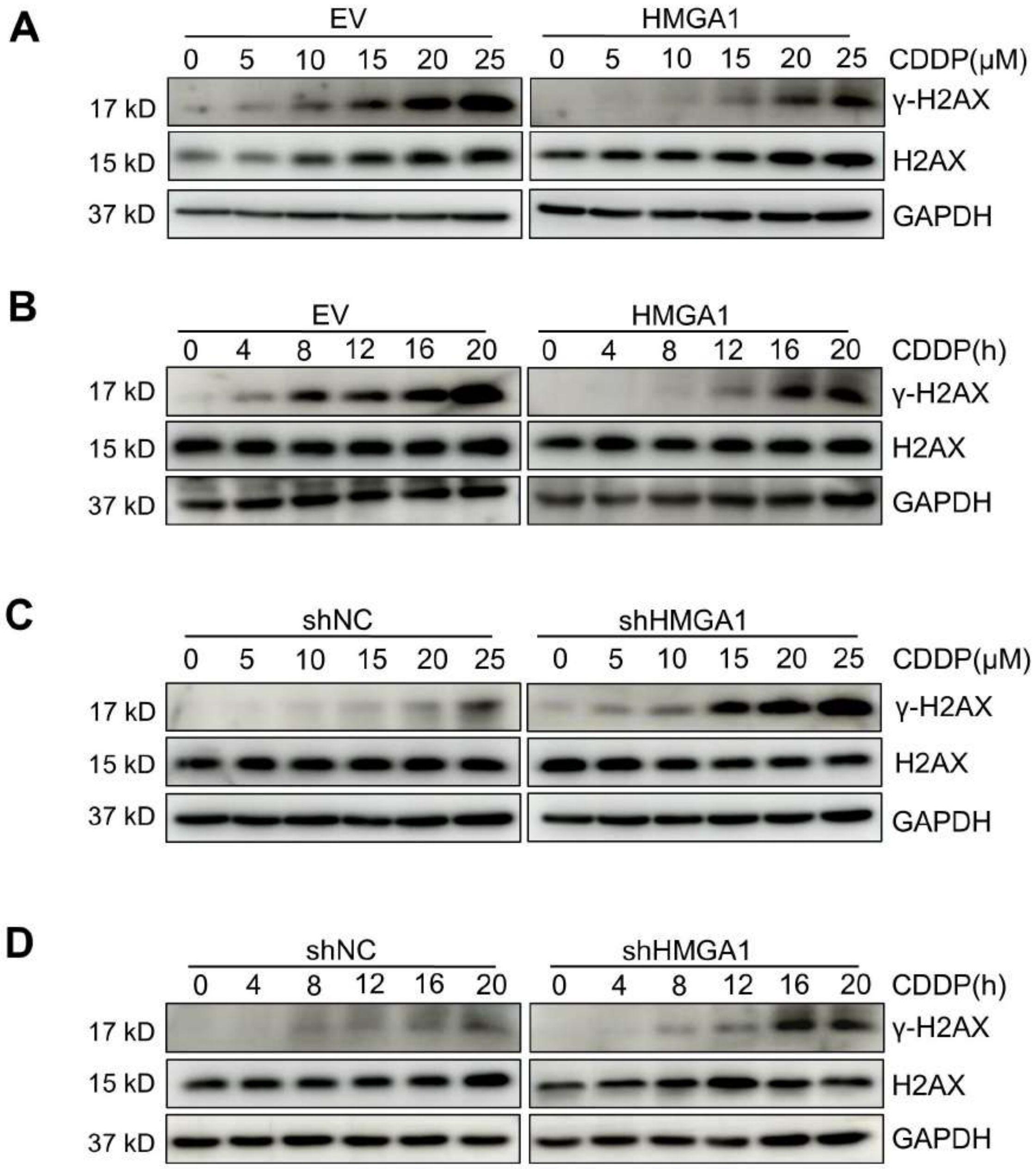
A
EV
HMGA1
0 5 10 15 20 25
0 5 10 15 20 25
CDDP(μM)
17 kD
15 kD
37 kD
γ-H2AX
H2AX
GAPDH
B
EV
HMGA1
0 4 8 12 16 20
0 4 8 12 16 20
CDDP(h)
17 kD
15 kD
37 kD
γ-H2AX
H2AX
GAPDH
C
shNC
shHMGA1
0 5 10 15 20 25
0 5 10 15 20 25
CDDP(μM)
17 kD
15 kD
37 kD
γ-H2AX
H2AX
GAPDH
D
shNC
shHMGA1
0 4 8 12 16 20
0 4 8 12 16 20
CDDP(h)
17 kD
15 kD
37 kD
γ-H2AX
H2AX
GAPDH

Supplementary Fig. 4 Alterations of γ-H2AX in HMGA1-manipulated KYSE30 and TE13 cells. A. The TE13 cells with or without HMGA overexpression were treated with different concentrations of CDDP for 16 h and the cell lysates were analyzed by western blotting (WB). **B.** The TE13 cells with or without HMGA overexpression were treated with CDDP (10 μM) for different time, and the cell lysates were analyzed by WB. **C.** The KYSE30 cells with or without HMGA knockdown were treated with different concentrations of CDDP for 16 h and the cell lysates were analyzed by WB. **D.** The KYSE30 cells with or without HMGA knockdown were treated with CDDP (10 μM) for different time, and the cell lysates were analyzed by WB.

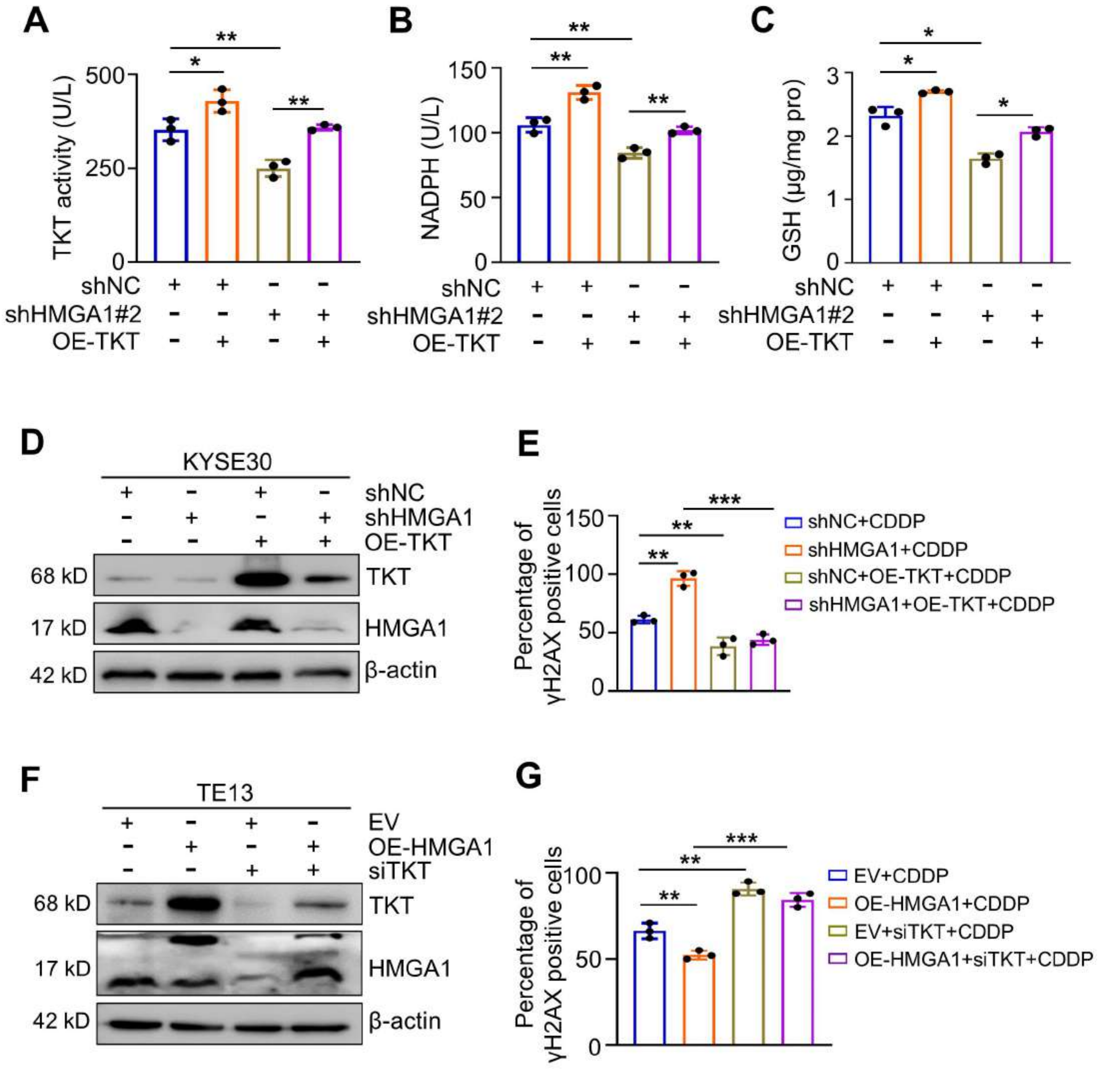
A
TKT activity (U/L)
500
250
0
shNC
shHMGA1#2
OE-TKT
B
NADPH (U/L)
150
100
50
0
C
GSH (μg/mg pro)
3
2
1
0
D
KYSE30
shNC
shHMGA1
OE-TKT
68 kD
TKT
17 kD
HMGA1
42 kD
β-actin
E
Percentage of γH2AX positive cells
150
100
50
0
shNC+CDDP
shHMGA1+CDDP
shNC+OE-TKT+CDDP
shHMGA1+OE-TKT+CDDP
F
TE13
EV
OE-HMGA1
siTKT
G
EV+CDDP
OE-HMGA1+CDDP
EV+siTKT+CDDP
OE-HMGA1+siTKT+CDDP

Supplementary Fig. 5 TKT mediates HMGA1-upregulated PPP. A - C. Control and HMGA1-knocked down KYSE30 cells were transfected with Flag-tagged TKT. TKT enzyme activity (**A**), NADPH (**B**), and GSH (**C**) were measured. **D.** Control and HMGA1-knocked down KYSE30 cells were transfected with pcDNA3.1/TKT. Western blotting was used for determining the expression of HMGA1 and TKT. **E.** Immunofluorescence was performed for the detection of γ-H2AX in cells treated with 10 μM CDDP for 16 h. The percentage of γ-H2AX-positive ESCC cells was calculated. Only cells with more than 5 γ-H2AX lesions are considered positive. The percentage of γ-H2AX-positive cells is quantified based on DNA repair activity. Count at least 100 cells per sample per experiment. **F.** Control and HMGA1-overexpressed TE13 cells were transfected with TKT siRNA. Western blotting was used for determining the expression of HMGA1 and TKT. **G.** Immunofluorescence was performed for the detection of γ-H2AX in cells treated with TKT siRNA and 10 μM CDDP. The experiment was performed as described in (**E**). Data are presented as the means ± S.D., and significant differences are indicated as * $P < 0.05$, ** $P < 0.01$, and *** $P < 0.001$, n = 3.
