# Supplementary figures and images for: HMGA1 promotes the progression of esophageal squamous cell carcinoma by elevating TKT-mediated upregulation of pentose phosphate pathway
Source: Cell Death Dis. 2024 Jul 30;15(7):541. doi: 10.1038/s41419-024-06933-x (PMC11289123; doi:10.1038/s41419-024-06933-x)

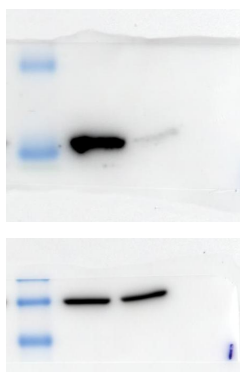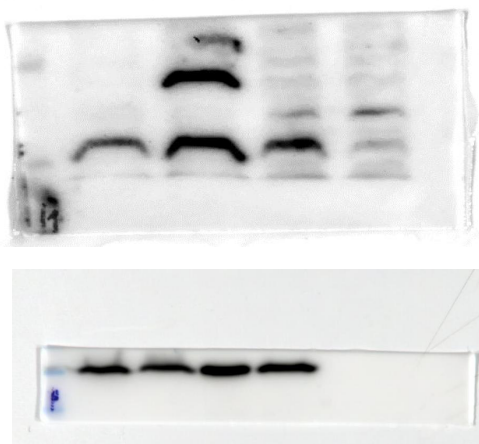

**A**

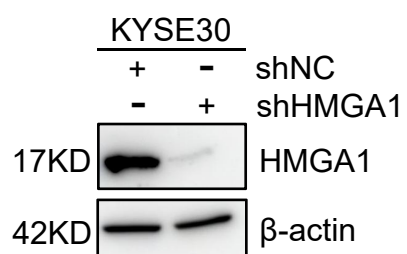

**G**

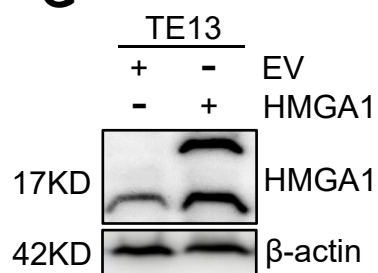

**Fig 2**

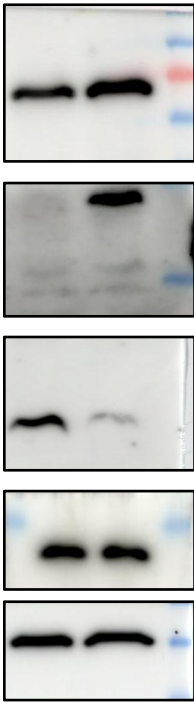

F

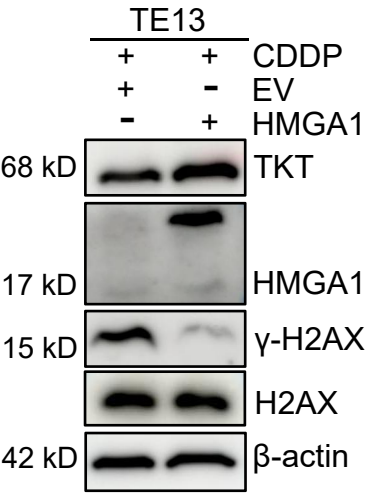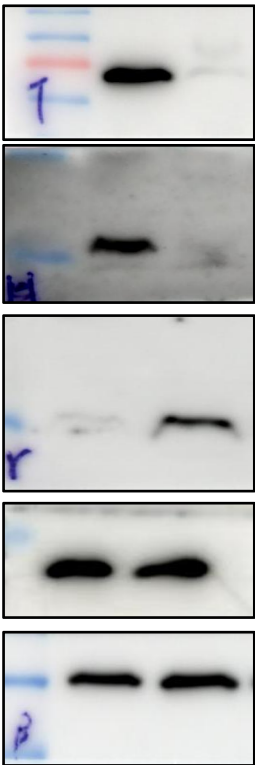

I

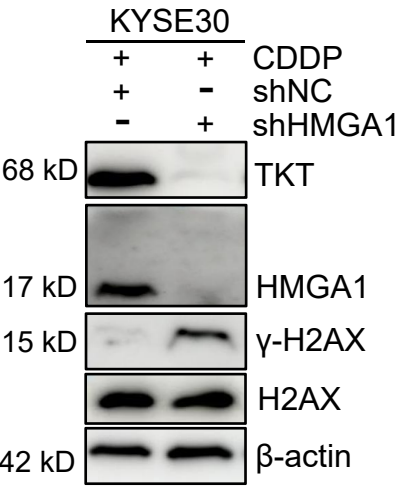

Fig 4

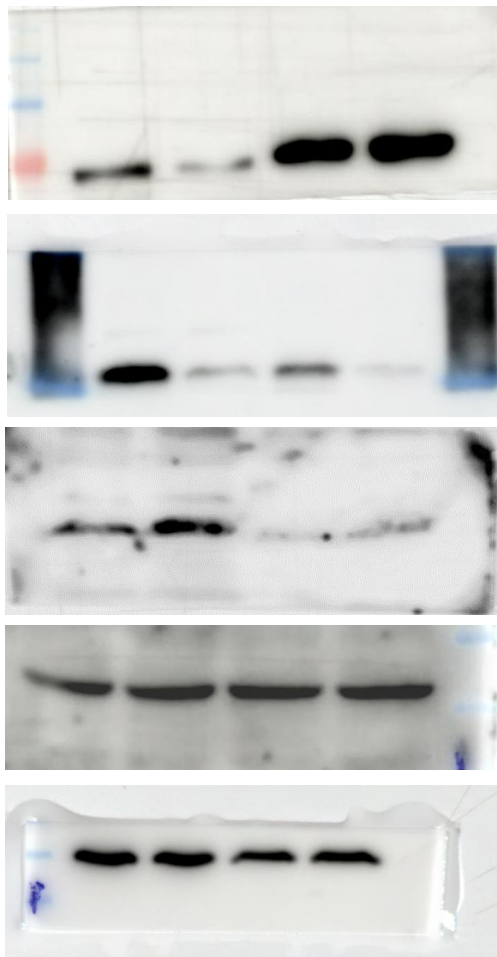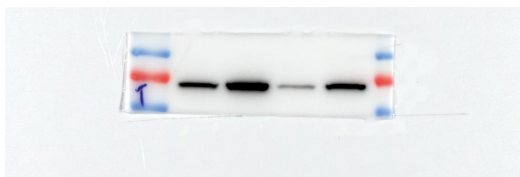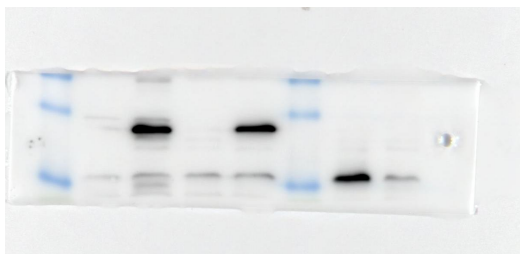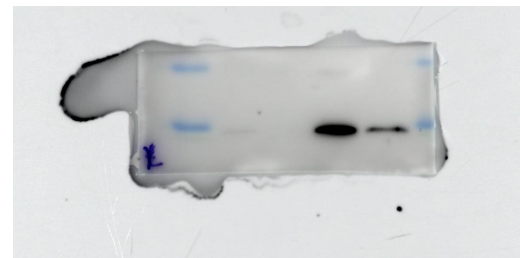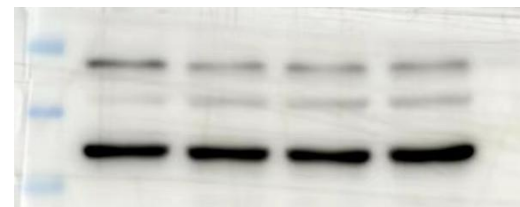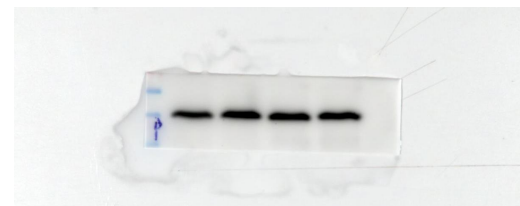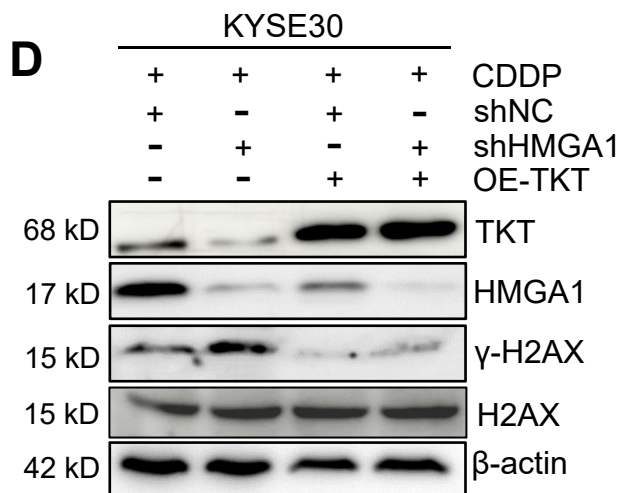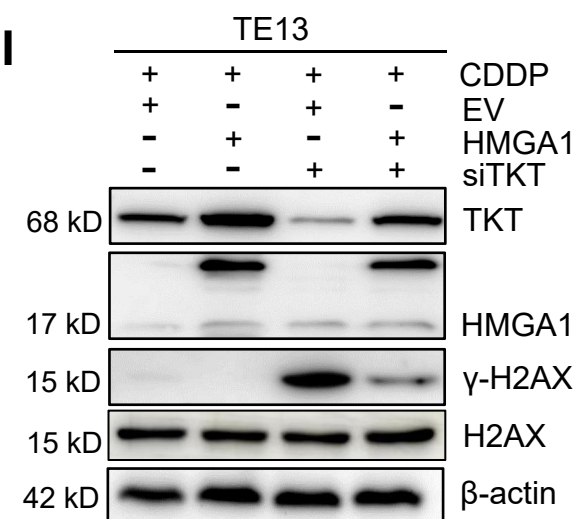

**Fig 5**

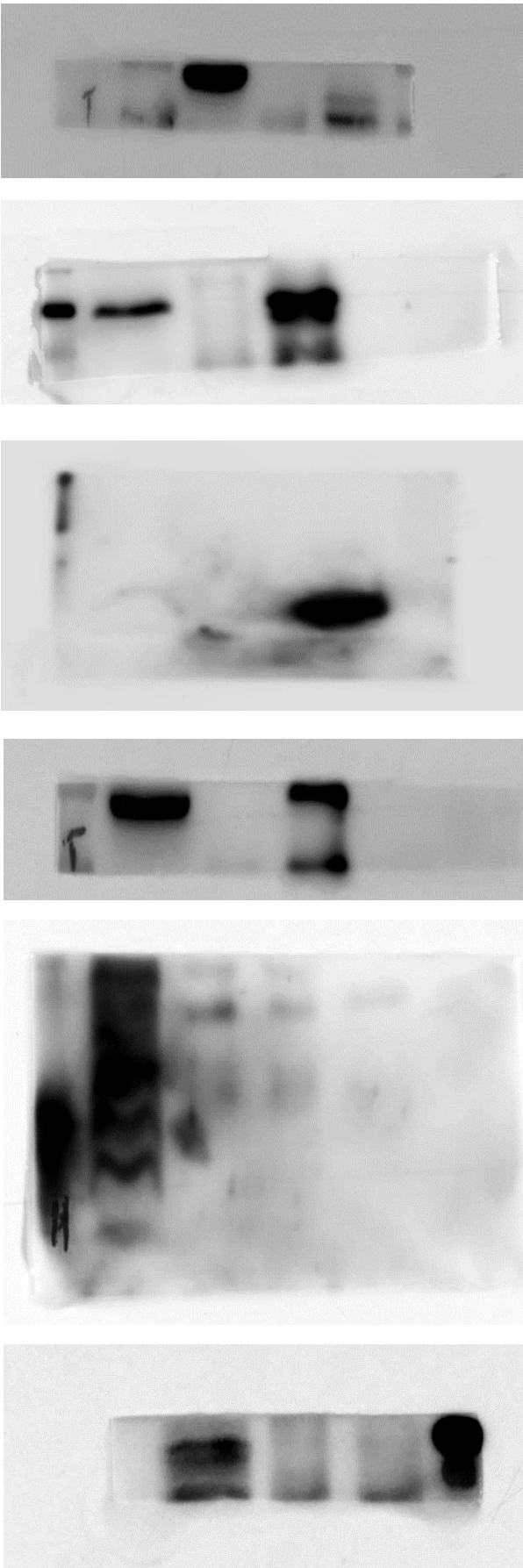

**A**

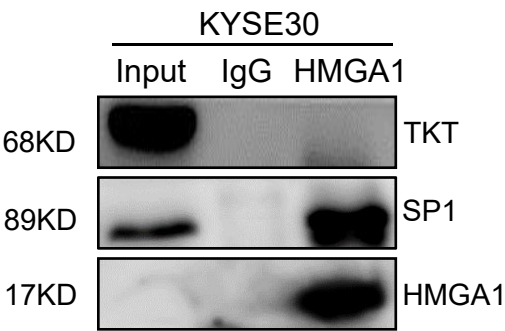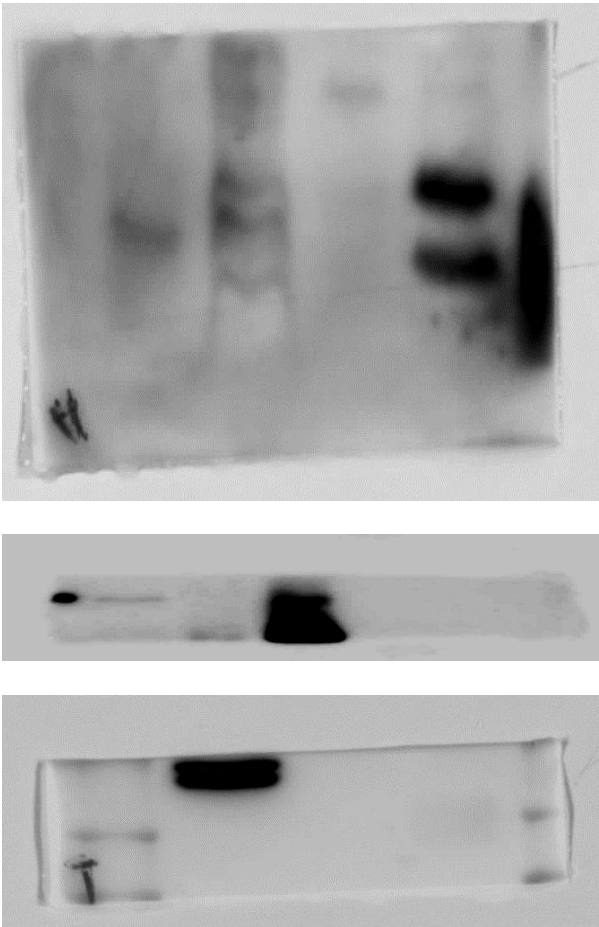

**B**

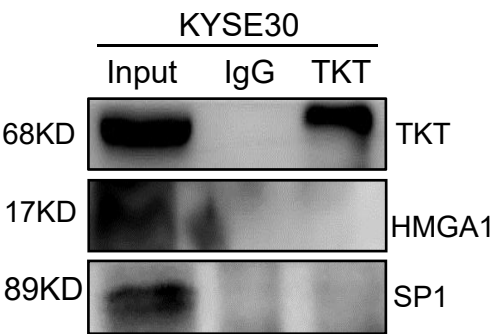

**C**

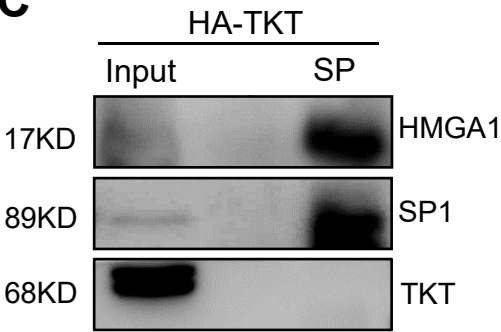

**Fig 6**

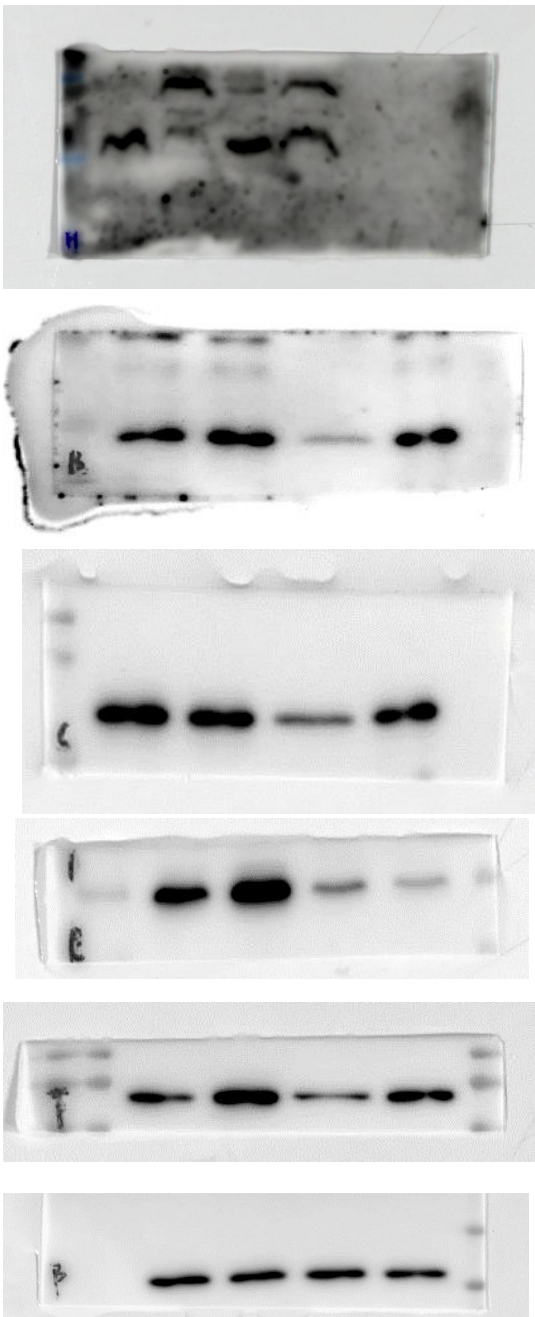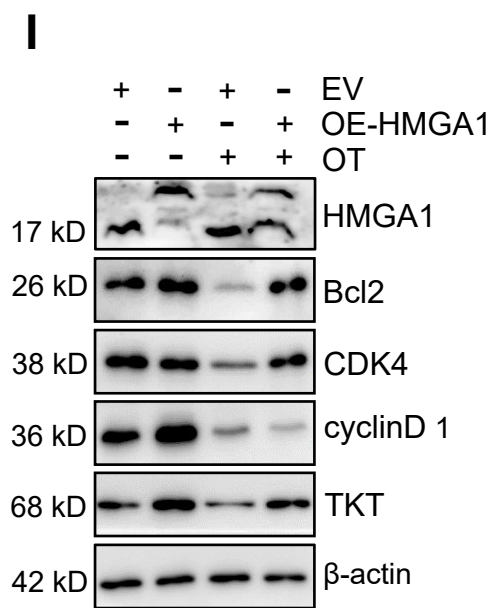

**Fig 7**

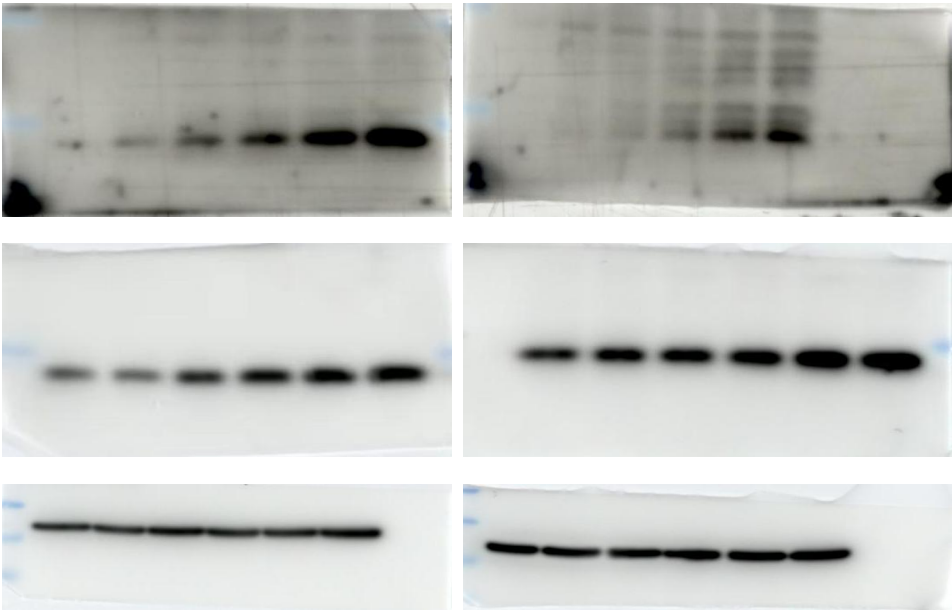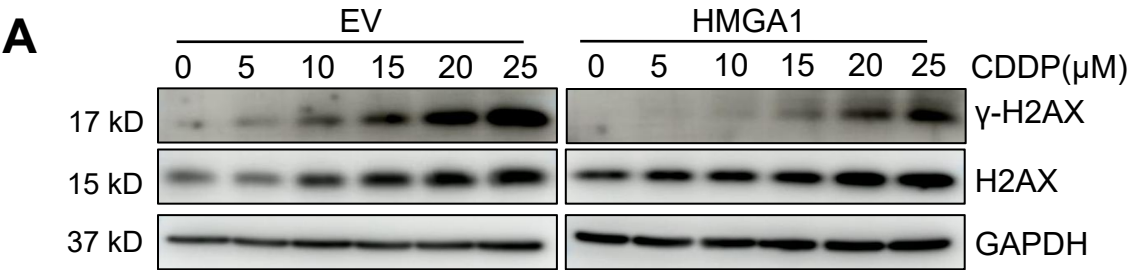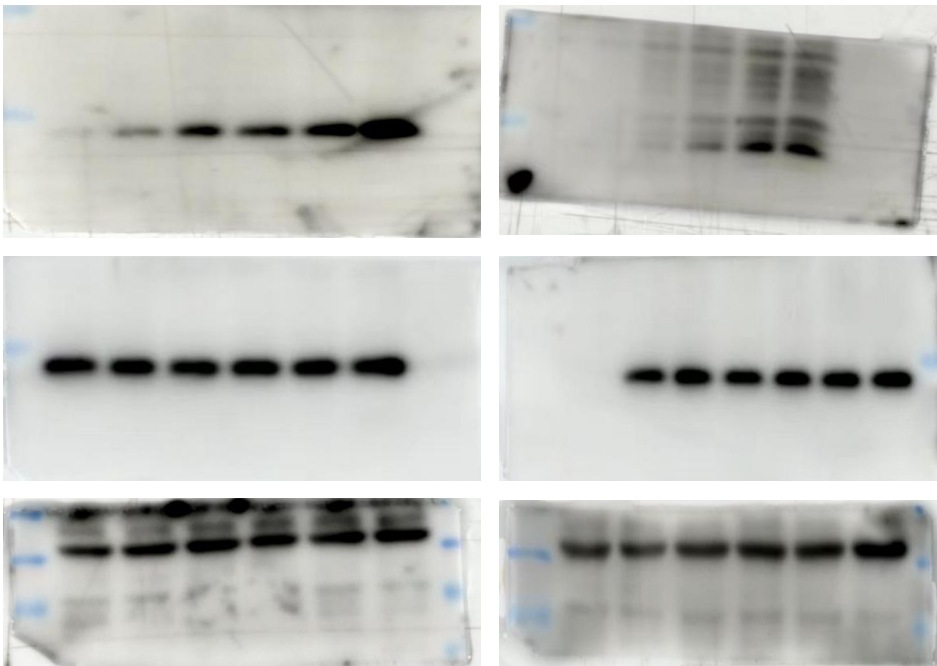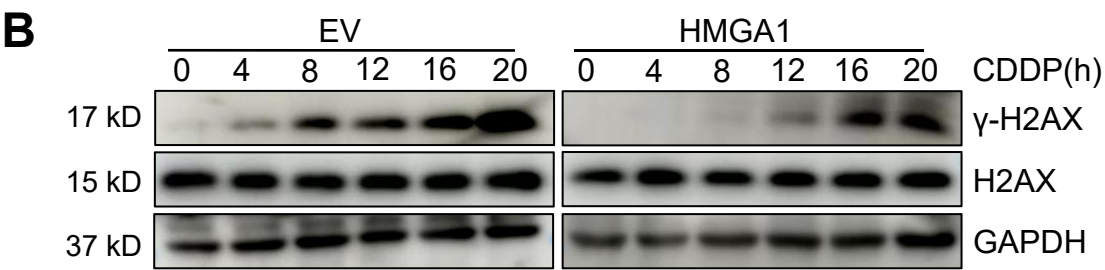

**Fig S4**

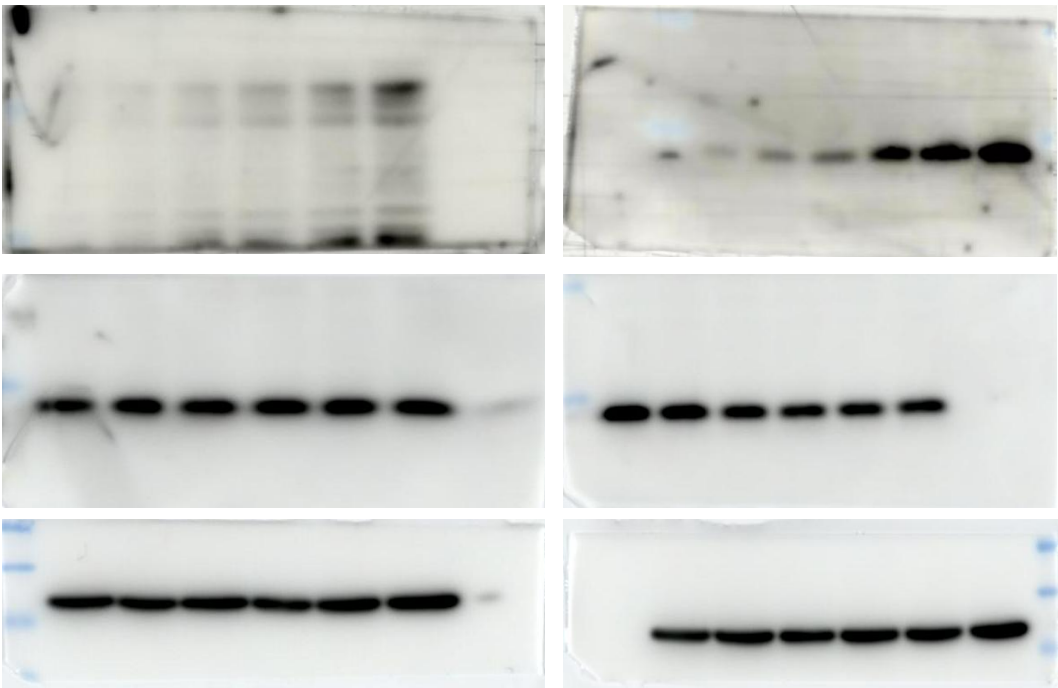

**C**

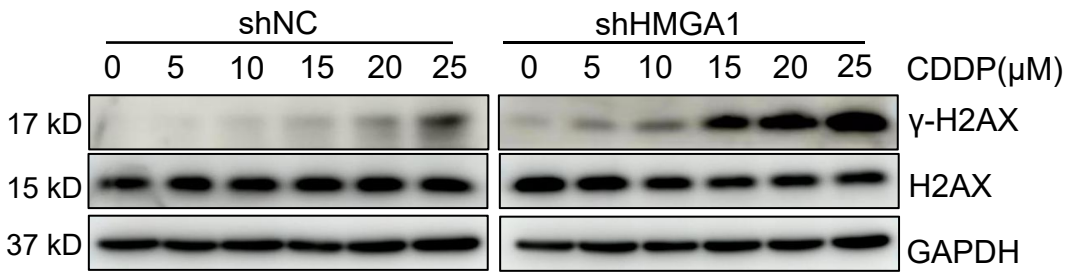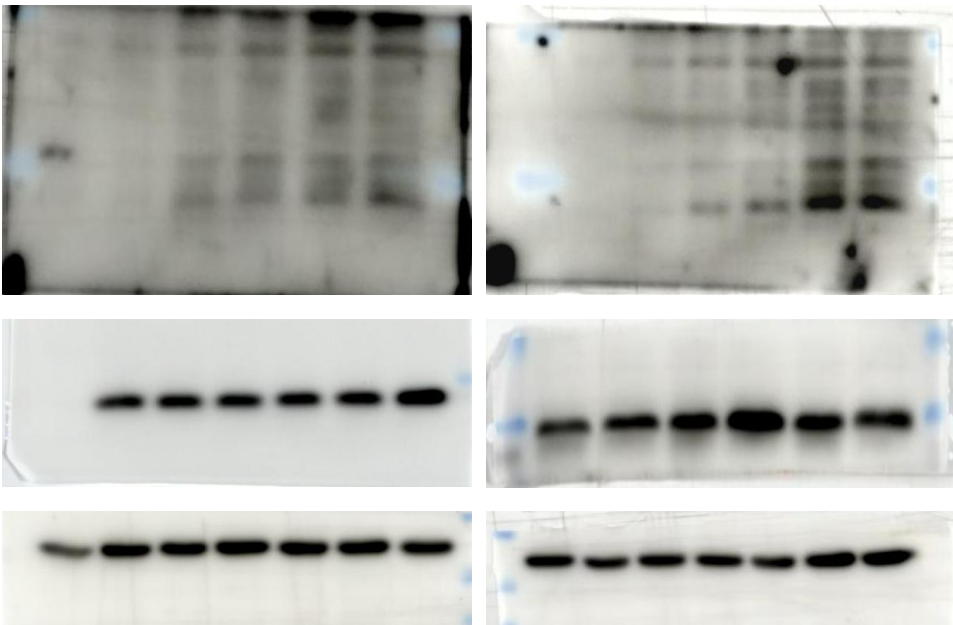

**D**

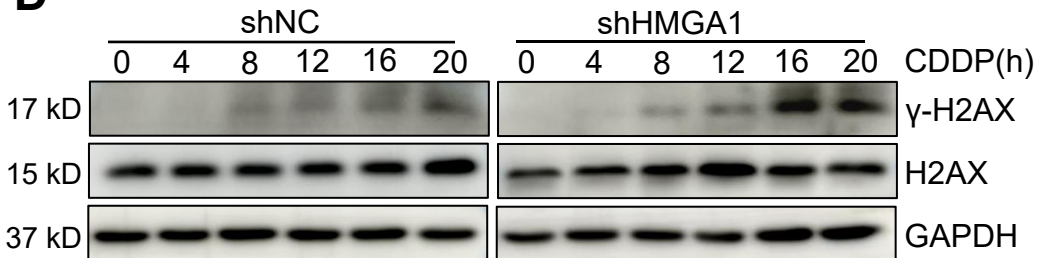

**Fig S4**

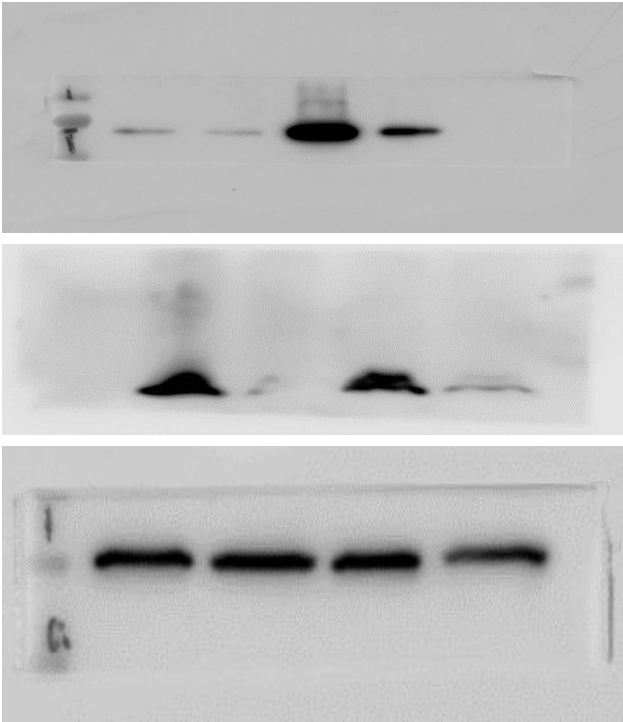

**D**

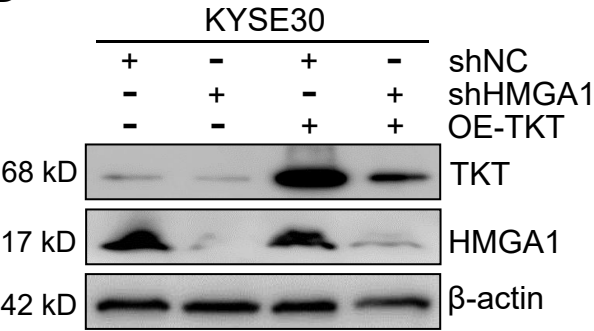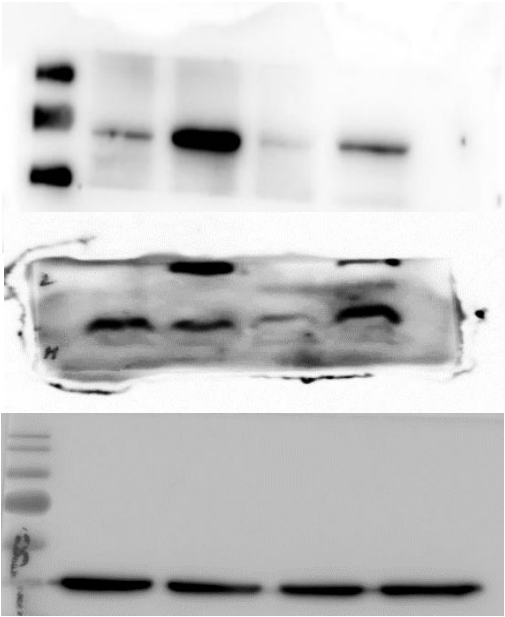

**F**

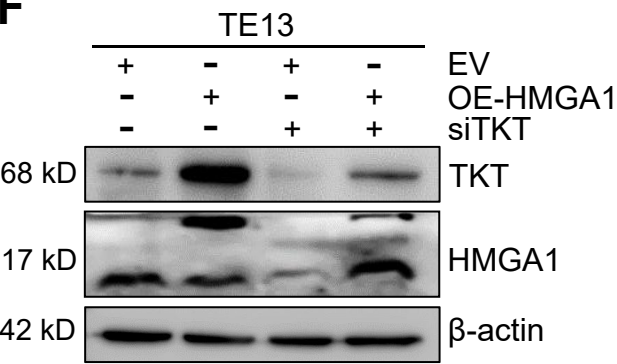

**Fig S5**

Supplement: Supplementary file 2 — Original Data [file 41419_2024_6933_MOESM2_ESM.pdf]
